# USP18 positively regulates innate antiviral immunity by promoting K63-linked polyubiquitination of MAVS

**DOI:** 10.1038/s41467-021-23219-4

**Published:** 2021-05-20

**Authors:** Jinxiu Hou, Lulu Han, Ze Zhao, Huiqing Liu, Lei Zhang, Chunhong Ma, Fan Yi, Bingyu Liu, Yi Zheng, Chengjiang Gao

**Affiliations:** 1 Key Laboratory of Infection and Immunity of Shandong Province & Department of Immunology, School of Basic Medical Sciences, Shandong University, Jinan, Shandong P. R. China; 2 Department of Pharmacology, School of Basic Medical Sciences, Shandong University, Jinan, Shandong P. R. China

**Keywords:** Infection, RIG-I-like receptors, Virology

## Abstract

Activation of MAVS, an adaptor molecule in Rig-I-like receptor (RLR) signaling, is indispensable for antiviral immunity, yet the molecular mechanisms modulating MAVS activation are not completely understood. Ubiquitination has a central function in regulating the activity of MAVS. Here, we demonstrate that a mitochondria-localized deubiquitinase USP18 specifically interacts with MAVS, promotes K63-linked polyubiquitination and subsequent aggregation of MAVS. USP18 upregulates the expression and production of type I interferon following infection with Sendai virus (SeV) or Encephalomyocarditis virus (EMCV). Mice with a deficiency of USP18 are more susceptible to RNA virus infection. USP18 functions as a scaffold protein to facilitate the re-localization of TRIM31 and enhances the interaction between TRIM31 and MAVS in mitochondria. Our results indicate that USP18 functions as a post-translational modulator of MAVS-mediated antiviral signaling.

## Abstract (web summary)

Ubiquitination has an important function in the regulation of antiviral immunity involving the signalling molecule MAVS. Here the authors investigate deubiquitinating enzymes and show USP18 regulates MAVS mediated antiviral signalling through modulating the ubiquitination of MAVS via promotion of interaction between MAVS and TRIM31.

## Introduction

Pathogen associated molecular patterns (PAMPs), such as viral nucleic acids, can be detected by the pattern recognition receptors (PRRs) of the host cell^1^. This interaction between viral PAMPs and cellular PRRs elicits an elegant innate immune signaling transduction. Viral DNA and RNA are recognized by different sensor proteins. The recognition of viral DNA by cyclic GMP-AMP synthase (cGAS) leads to the production of the second messenger cyclic [G(2′,5′)pA(3′,5′)p] (2′3′-cGAMP), which is the agonist of downstream adaptor protein stimulator of interferon genes (STING)^2^. The sensing of viral RNA by RLRs (RIG-I-like receptors) results in their activation and subsequent interaction with the adaptor protein mitochondrial antiviral signaling protein (MAVS)^3^. The two pathways converge on the downstream kinases and effector proteins. The activation of STING and MAVS leads to the phosphorylation of downstream kinase TBK1, which phosphorylate IRF3. The phosphorylated IRF3 dimerizes and translocates into the nucleus to stimulate the expression of type-I interferon, which is critical for controlling the spread of viral infection.

In the RLRs pathway, binding of cytosolic RNA with RIG-I and MDA5 leads to the unmasking and oligomerization of their CARD domain^3^. The activated CARD domain of RLRs further interacts with the CARD domain of mitochondrial protein MAVS, resulting in the formation of the prion-like structure of MAVS^4^. This process is dependent on the CARD domain of RIG-I and MAVS as well as unanchored ubiquitin chains^5^. Aggregation of MAVS is the hallmark of its activation and required for the downstream antiviral signaling transduction. Multiple proteins have been reported to modulate the aggregation of MAVS. SNX8 positively regulates the formation of MAVS aggregates, while ATG5 and COX5B inhibit this process^6,7^. Besides, M2 protein from the influenza virus has also been reported to regulate the aggregation of MAVS^8^. These studies suggest that the prion-like formation of MAVS is a critical process that needs to be tightly controlled in antiviral innate immunity. In addition to its importance in antiviral immunity, several studies have suggested the correlation between MAVS aggregates with higher type-I interferon in systemic lupus erythematosus (SLE)^9^. Recently, it has been reported that multiple truncated isoforms of MAVS limit its spontaneous aggregation^10^. These studies implicate that aggregation of MAVS must be tightly controlled to prevent an overwhelming immune response.

A series of studies have emphasized the importance of post-translational modification (PTM) in modulating the activity of MAVS^11^. Especially, the studies from our lab and other labs have emphasized that ubiquitination is indispensable for regulating the activation and stability of MAVS^12^. There are abundant numbers of E3 ubiquitin ligases that are responsible for K63-, K27-, and K48-linked polyubiquitination of MAVS, such as TRIM31, TRIM21, and Smurf1^12^. Since different ubiquitin linkages are correlated with distinct functions, these E3 ligases play distinctive roles in antiviral innate immunity. Our lab has identified that the E3 ligase TRIM31 is responsible for the K63-linked polyubiquitination of MAVS critical for the aggregation of MAVS^13^, while K27-linked polyubiquitination catalyzed by TRIM21 facilitates the interaction between MAVS and TBK1^14^. K48-linked ubiquitin chains are canonical signals for the degradation of proteins. Therefore, Smurf1 facilitates the turnover of MAVS during viral infection^15^. Ubiquitination is a reversible process maintained by both E3 ligases and deubiquitinases (DUBs). Therefore, DUBs also play significant roles in modulating the turnover and activation of MAVS^16^. OTUD1 and OTUD4 regulate the turnover of MAVS in different manners. OTUD1 promotes the stability of E3 ligase Smurf1 through removing its K48-linked polyubiquitin chains. Eventually, OTUD1 affect the antiviral immune response by enhancing the turnover of MAVS^17^. Different from OTUD1, OTUD4 directly deconjugates the K48-linked polyubiquitin chains from MAVS to enhance its stability^18^. YOD1 re-localizes to mitochondria upon viral infection and cleaves the K63-linked polyubiquitin chains from MAVS^19^. These studies suggest a fine-tuned regulation of MAVS activity by ubiquitination in viral infection. However, how these E3 ubiquitin ligases and DUBs cooperate to modulate the ubiquitination level of MAVS in a timely and spatial manner warrants further investigation.

Ubiquitin-specific peptidase 18 (USP18) is a multifunctional protein^20^. Originally, USP18 is identified as the deISGlase and removes interferon-stimulated gene 15 (ISG15) from the substrate proteins^21^. ISG15 has been implicated to plays an important role during host responses to viral infections. Consistent with this notion, Usp18^C61A^ cells are more resistant to influenza B virus infection compared with wild-type cells after type I IFN treatment, and deletion of ISG15 in Usp18^C61A^ background fully restores viral replication^22^. Apart from its enzymatic function, USP18 is also known as the negative inhibitor of the type-I interferon signaling through competing with JAK for interaction with IFNAR^23^. Consequently, the treatment of cells bearing the deletion of USP18 with type-I interferon leads to prolonged phosphorylation of STAT1 compared with the wild-type counterparts, which is consistent with the phenomenon that suppression of USP18 can potentiate the anti-HBV activity of interferon-α in HepG2.2.15 cells^24^. A recent study has suggested that USP18 recruits another DUB USP20 to deconjugate the K33- and K48-linked polyubiquitin chains from STING^25^. Subsequently, the deficiency of USP18 led to the increased turnover of STING and impaired type-I interferon response following DNA virus infection. Furthermore, this study found that the antiviral ability of Usp18^C61A^ cells was comparable to wild-type cells against herpes simplex virus 1(HSV-1) infection, suggesting the enzymatic activity of USP18 is dispensable. Recently, several studies also indicate that USP18 acts as a direct DUB and deconjugates the K63-linked polyubiquitin chains from TAK1^26^. These studies suggest that USP18 is closely associated with different signaling pathways through both enzymatic-dependent or independent manners.

In this study, we utilize Psort Wolf software to predict the DUBs potentially found in mitochondria. We find 13 DUBs that are likely to be located in mitochondria. We observe that USP18 can significantly promote the polyubiquitination of MAVS. The interaction between USP18 and MAVS is enhanced following viral infection, suggesting the possible regulation of MAVS activity by USP18. Furthermore, USP18 facilitates the K63-linked polyubiquitination and subsequent aggregation of MAVS. Consequently, deletion of USP18 in macrophages or fibroblasts leads to impaired type-I interferon response following viral infection. Mice with a deficiency of USP18 are more susceptible to viral infection. The effect of USP18 on MAVS is independent of its enzymatic activity but dependent on TRIM31. Upon viral infection, USP18 is critical for the re-localization of TRIM31 from the cytoplasm to mitochondria and the interaction between TRIM31 and MAVS.

## Results

### USP18 enhances MAVS polyubiquitination

**Fig. 1:**
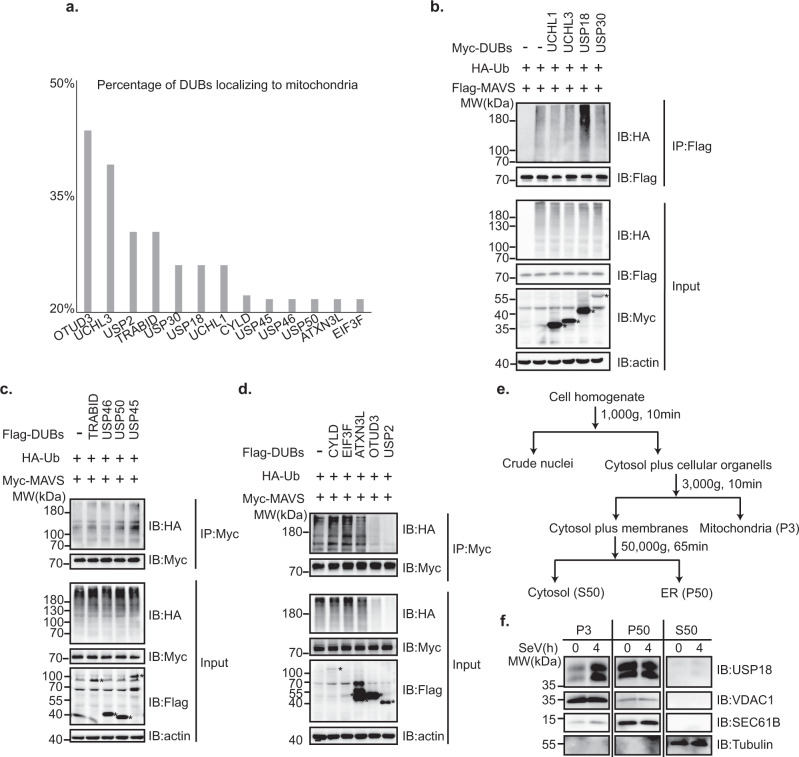
Identification of USP18 that regulates the polyubiquitination of MAVS. **a** Illustration of DUBs likely to be distributed in mitochondria. The bar graph illustrates the DUBs with >20% possibility present in mitochondria according to the prediction of Psort Wolf software. **b**–**d** Immunoprecipitation analysis of ubiquitination of MAVS in HEK293T cells transfected with the plasmids expressing Flag-MAVS (**b**) or Myc-MAVS (**c**, **d**), HA vector or HA-WT ubiquitin (Ub), together with indicated DUBs. After 24 h of transfection, the cells were lysed and immunoprecipitated with Anti-Flag magnetic beads (**b**) or Anti-Myc magnetic beads (**c**, **d**). Cell lysates and immunoprecipitates were analyzed by immunoblot with indicated antibodies. Actin was used as the loading control throughout. **e** Schematic diagram to isolate the mitochondria and ER fraction. **f** Immunoblot analysis of USP18 from the mitochondrial (P3), ER (P50), and cytoplasmic (S50) fraction of THP-1 infected with SeV for indicated time points. VDAC1, SEC61B, and tubulin were used as the mitochondrial, ER, and cytosolic markers, respectively. Asterisk was marked right next to the band corresponding to the DUBs in Fig. 1b–d.

Since MAVS is specifically localized in the outer membrane of mitochondria and is extensively regulated by ubiquitination modification, we speculated DUBs present in mitochondria could cooperate with E3 ubiquitin ligase to tightly regulate the ubiquitination level of MAVS. We utilized the Psort Wolf software (https://wolfpsort.hgc.jp/) to predict the possibility of DUBs in mitochondria. Through inputting the protein sequence of ~100 DUBs, we found that 13 DUBs are likely distributed in mitochondria over the threshold of 20% (Fig. 1a). USP30, one of the well-known mitochondria-localized DUBs^27^, was predicted to be present in mitochondria in our study, suggesting the accuracy of our methodology. To search for the DUBs involved in the modulation of MAVS ubiquitination, we co-transfected the plasmids encoding HA-ubiquitin (Ub), MAVS, and 13 DUBs into HEK293T cells. The polyubiquitination level of MAVS was examined by probing the immunoprecipitates with the HA antibody. Interestingly, we found USP18 significantly enhanced, rather than reduced, the polyubiquitination level of MAVS compared to other DUBs (Fig. 1b). For the rest of the DUBs, we observed that both OTUD3 and USP2 decreased the polyubiquitination level of MAVS (Fig. 1c, d). However, the input HA-Ub level of total cell lysate was also significantly declined, suggesting OTUD3 and USP2 likely removed the ubiquitin from a broad range of substrates.

A previous study has suggested that USP18 is an interferon-inducible gene and stimulated by LPS^28^. We examined USP18 expression during RNA virus infection and found that SeV infection greatly increased the human USP18 mRNA and USP18 protein in human THP-1 cells (Supplementary Fig. 1a, b). Similarly, we observed that the level of mRNA and protein of USP18 was increased in primary peritoneal macrophages (PMs) following SeV infection (Supplementary Fig. 1c, d). A previous study has suggested that USP18 is present in mitochondria through binding to BCL2L1 in HCC cells^29^. To investigate whether USP18 is associated with mitochondria, we isolated the mitochondria from THP-1 cells according to the protocol from a previous study^30^ (Fig. 1e) and found that USP18 was indeed enriched in the mitochondrial fraction upon SeV infection (Fig. 1f), which is consistent with the prediction results from Psort Wolf software. More importantly, we observed that the protein level of USP18 was enhanced in mitochondria rather than ER following RNA virus infection (Fig. 1f). These data demonstrated that USP18 is a mitochondrion-associated protein, likely involved in the regulation of antiviral innate immunity through promoting MAVS ubiquitination.

### USP18 regulates IFN-β signaling upon RNA virus infection

Next, we investigated the physiological role of USP18 in innate immunity against RNA virus infection. To explore the potential role of USP18 in antiviral signaling, we first designed a small interfering RNA (siRNA) that targeted human *USP18* and transfected it into human THP-1 cells. We found that expression of endogenous mRNA and protein level of USP18 was much lower in cells transfected with the USP18-specific siRNA than in those transfected with control (non-targeting) siRNA (Supplementary Fig. 2a). siRNA knockdown of *USP18* expression significantly decreased the expression of *IFNB* mRNA and downstream *CCl5* mRNA in THP-1 cells after SeV infection (Supplementary Fig. 2b). Akin to the data obtained with THP-1 cells, we observed that the siRNA knockdown of *Usp18* expression in primary mouse macrophages decreased SeV-induced expression of *Ifnb* and production of IFN-β (Supplementary Fig. 2c). Furthermore, the siRNA-mediated knockdown of mouse *Usp18* expression in mouse macrophages also decreased EMCV-induced expression of *Ifnb* and production of IFN-β (Supplementary Fig. 2d). Since SeV and EMCV are recognized by RIG-I and MDA5 respectively, this phenomenon indicated that USP18 regulates both RIG-I and MDA5-mediated innate antiviral signaling.

**Fig. 2:**
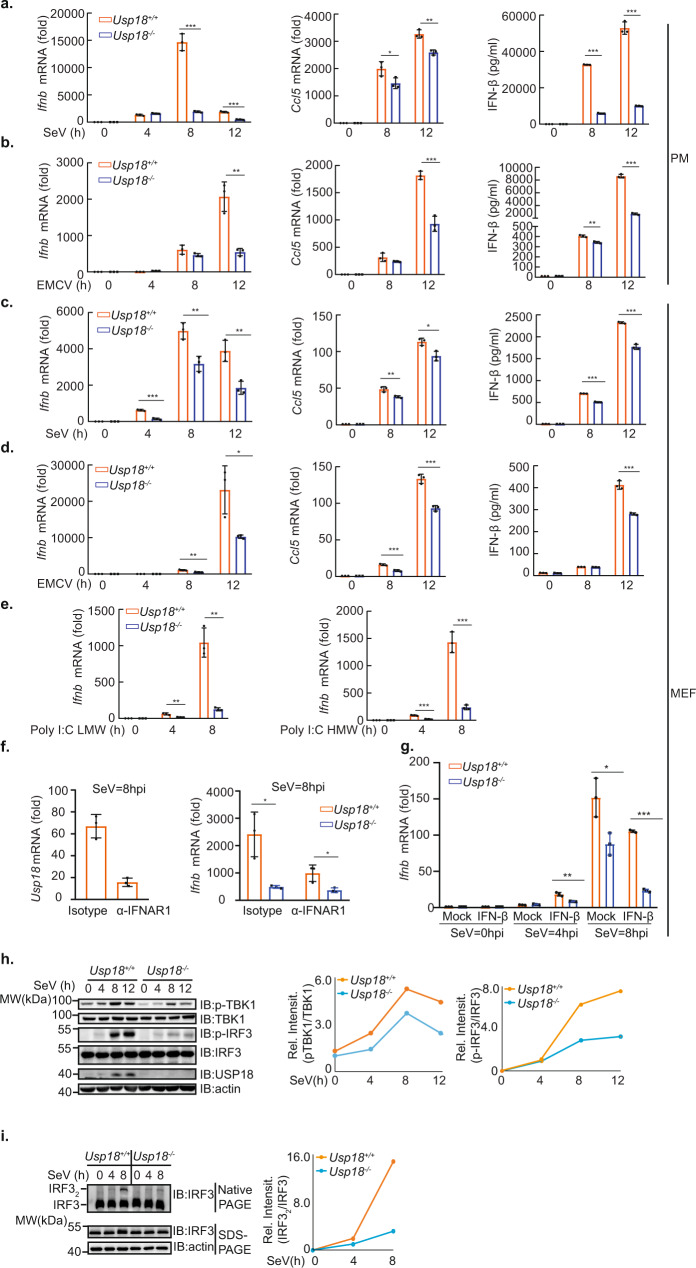
USP18 positively regulates RLR-induced IFN-β signaling upon RNA virus infection. **a**, **b** qRT-PCR analysis of *Ifnb* (left), *Ccl5* (middle), and ELISA analysis of IFN-β (right) from the culture supernatant of *Usp18*^*+/+*^ and *Usp18*^*−/−*^ peritoneal macrophages infected with SeV (**a**) or EMCV (**b**) for indicated time points. (Representative data were collected and expressed as mean ± SD from three independent experiments. Two-tailed Student’s *t* test was performed, For **a**, left panel: ***p < 0.001, ***p < 0.001 in sequence, middle panel: *p = 0.0477, **p = 0.0029 in sequence, right panel: ***p < 0.001, ***p < 0.001 in sequence; For **b**, left panel: **p = 0.0032, middle panel: ***p < 0.001 in sequence, right panel: **p = 0.0013, ***p < 0.001 in sequence). **c**, **d** qRT-PCR analysis of *Ifnb* (left), *Ccl5* (middle) mRNA, and ELISA analysis of IFN-β (right) from the culture supernatant of *Usp18*^*+/+*^ and *Usp18*^*−/−*^ MEFs infected with SeV (**c**) or EMCV (**d**) for indicated time points. (Representative data were collected and expressed as mean ± SD from at least three independent experiments. Two-tailed Student’s *t* test was performed, For **c**, left panel: ***p < 0.001, **p = 0.0069, *p = 0.0068 in sequence, middle panel: **p = 0.0096, *p = 0.0145 in sequence, right panel: ***p < 0.001, ***p < 0.001 in sequence; For **d**, left panel: **p = 0.0028, *p = 0.0281 in sequence, middle panel: ***p < 0.001, ***p < 0.001 in sequence, right panel: ***p < 0.001. **e** qRT-PCR analysis of *Ifnb* from *Usp18*^*+/+*^ and *Usp18*^*-/-*^ MEFs transfected with Poly I:C LMW (left) or Poly I:C HMW (right) for indicated time points. (Representative data were collected and expressed as mean ± SD from at least three independent experiments. Two-tailed Student’s *t* test was performed, left panel: **p = 0.0061, **p = 0.0014 in sequence, right panel: ***p < 0.001, ***p < 0.001 in sequence;) **f** Left panel, qRT-PCR analysis of *Usp18* from *Usp18*^*+/+*^ cells pretreated with isotype antibody or α-IFNAR1 antibody followed by SeV infection for 8 h. Right panel, qRT-PCR analysis of *Ifnb* in *Usp18*^*+/+*^ and *Usp18*^*−/−*^ cells pretreated with isotype antibody or α-IFNAR1 antibody followed by SeV infection for 8 h. (Representative data were collected and expressed as mean *±* SD from at least three independent experiments. Two-tailed Student’s *t* test was performed, *p = 0.0154, *p = 0.0274 in sequence). **g** qRT-PCR analysis of *Ifnb* in *Usp18*^*+/+*^ and *Usp18*^*−/−*^ MEF cells pretreated with or without mouse recombinant IFN-β followed by SeV infection for 8 h. (Representative data were collected and expressed as mean ± SD from at least three independent experiments. Two-tailed Student’s *t* test was performed, **p = 0.0025 *p = 0.0227 ***p < 0.001). **h** Immunoblot analysis of total and phosphorylated (p-) TBK1, total and phosphorylated (p-) IRF3 in lysates of *Usp18*^*+/+*^ and *Usp18*^*−/−*^ MEFs infected with SeV for 0–12 h and harvested at indicated time points. For the densitometric analysis (right), bands were normalized with individual actin, and phosphorylated protein over the total amount of that protein was determined. **i** Native gel analysis (above) and SDS-PAGE (below) of IRF3 dimerization in lysates of *Usp18*^*+/+*^ and *Usp18*^*−/−*^ MEFs infected with SeV for 0–8 h. Densitometry of dimer IRF3 versus IRF3 (right). Representative data were collected and expressed as mean ± SD from at least three independent experiments. Two-tailed Student’s *t* test was performed, with *p < 0.05, **p < 0.01, ***p < 0.001 for *Usp18*^*+/+*^ versus *Usp18*^*−/−*^ (**a**–**g**).

Next, we prepared primary peritoneal macrophages from *Usp18*^*+/+*^ and *Usp18*^*−/−*^ mice. Consistent with the observation from the siRNA knockdown of USP18, infection of *Usp18*^*−/−*^ macrophages with SeV led to a decrease in fold changes of *Ifnb* mRNA as well as the production of IFN-β compared with *Usp18*^*+/+*^ macrophages (Fig. 2a). Congruently, the fold changes of *Ccl5* mRNA from *Usp18*^*−/−*^ peritoneal macrophages were also declined compared with *Usp18*^*+/+*^ counterparts (Fig. 2a). Infection of *Usp18*^*−/−*^ peritoneal macrophages with EMCV also led to a decrease in fold change of *Ifnb* and its downstream *Ccl5* gene mRNA levels as well as the production of IFN-β (Fig. 2b) compared with the WT counterparts. To further validate the effect of USP18 in other cell types, we isolated the primary MEFs from *Usp18*^*+/+*^ and *Usp18*^*−/−*^ mice and infected them with either SeV or EMCV. Akin to the phenomenon we observed in primary peritoneal macrophages, *Usp18*^*−/−*^ MEFs exhibited significantly impaired expression of *Ifnb* and its downstream genes as well as the production of IFN-β compared with *Usp18*^*+/+*^ MEFs (Fig. 2c, d). The positive regulation of USP18 in the RLR pathway was independent of RNA viral infection, since the deletion of USP18 in MEFs significantly impaired the expression of *Ifnb* after transfection of viral RNA analog Poly(I:C) LMW and HMW (Fig. 2e), which are the ligands for RIG-I and MDA5 respectively.

A previous study suggests that USP18 negatively regulates type-I interferon signaling by preventing the interaction between JAK and IFNAR^23^. Therefore, we next investigated whether the effect of USP18 on RLR-mediated type-I interferon response is related to IFNAR. To examine this, we pretreated the MEF cells with an anti-IFNAR antibody, which can efficiently block the induction of IFN downstream genes, as indicated by the much weaker induction of *Usp18* expression upon SeV infection (Fig. 2f, left panel). We observed that pretreatment with IFNAR antibody still resulted in a significant difference between *Usp18*^*+/+*^ and *Usp18*^*−/−*^ cells followed by SeV infection (Fig. 2f, right panel). However, we observed that the difference was smaller than that with isotype antibody treatment (Fig. 2f, right panel). Furthermore, we found that pretreatment of MEF cells with mouse recombinant IFN-β led to an enhanced difference between *Usp18*^*+/+*^ and *Usp18*^*−/−*^ cells following SeV infection (Fig. 2g). Altogether, these data suggest type I IFN and IFNAR partially contribute to the regulatory effect of USP18 on RLR-mediated signaling.

To assess changes in RIG-I and MDA5-mediated signaling cascades upon viral infection, we analyzed changes in the phosphorylation of TBK1 and IRF3, which are the downstream events of MAVS activation and hall marker of interferon pathway activation. Phosphorylated TBK1 (pTBK1) and IRF3 (pIRF3) were efficiently induced after SeV infection in *Usp18*^*+/+*^ MEFs, while these signals were significantly lower in *Usp18*^*−/−*^ MEFs (Fig. 2h). Since phosphorylated IRF3 led to the dimerization of IRF3, we then examined changes in the dimerization of IRF3. We observed that *Usp18*^*−/−*^ MEFs exhibited less dimerized IRF3 compared with *Usp18*^*+/+*^ MEFs (Fig. 2i). Altogether, these data indicated that USP18 positively regulates IFN-β signaling upon SeV and EMCV infection.

### USP18 regulates RNA virus infection

**Fig. 3:**
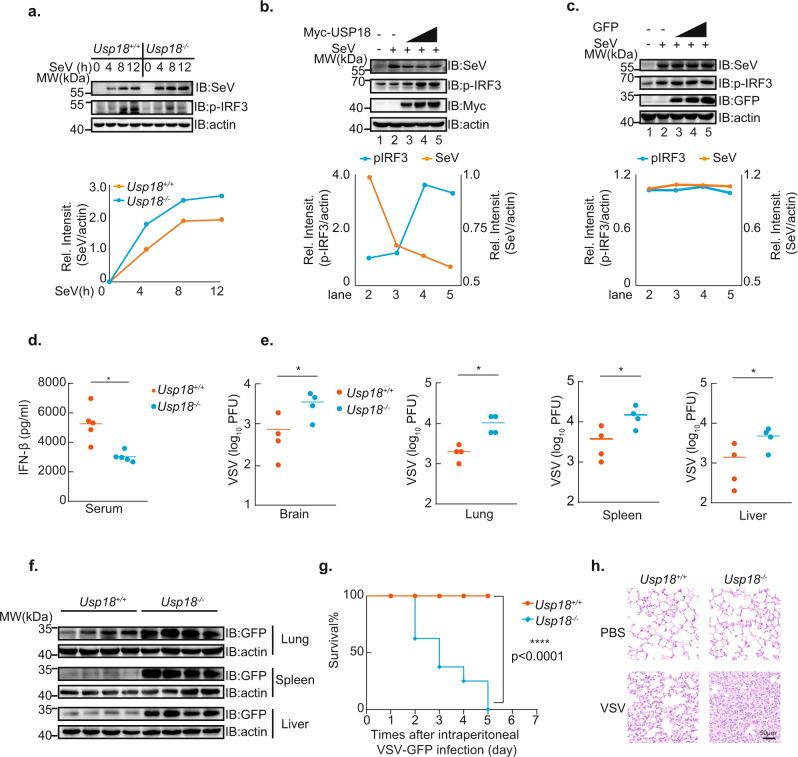
USP18 deficient mice are susceptible to RNA virus infection. **a** Immunoblot analysis of SeV in lysates of *Usp18*^*+/+*^ and *Usp18*^*−/−*^ MEFs infected with SeV for 0–12 h. **b** Immunoblot analysis of SeV in lysates of 293T cells transfected with Myc-USP18 in different dosages followed by infection with SeV for 8 h. **c** Immunoblot analysis of SeV in lysates of 293T cells transfected with EGFP-N1 plasmid in different dosages followed by infection with SeV for 8 h. **d** ELISA analysis of IFN-β level in serum of *Usp18*^*+/+*^ and *Usp18*^*−/−*^ mice (five per group) infected for 12 h by intraperitoneal injection of VSV (2 × 10^7^ PFU per mouse). Representative data were collected and expressed as mean ± SD from at least three independent experiments. Two-tailed Student’s *t* test was performed, *p = 0.0114. **e** Plaque assay of VSV titers in the brain, spleen, liver, and lungs of *Usp18*^*+/+*^ and *Usp18*^*−/−*^ mice (four per group) infected for 36 h by intraperitoneal injection of VSV (2 × 10^7^ PFU per mouse). Representative data were collected and expressed as mean ± SD from at least three independent experiments. Two-tailed Student’s *t* test was performed, *p = 0.0464, *p = 0.0178, *p = 0.0468, *p = 0.0428 in sequence. **f** Immunoblot analysis of VSV-GFP in the spleen, liver, and lungs of *Usp18*^*+/+*^ and *Usp18*^*−/−*^ mice (four per group) as in **e**. **g** Survival (Kaplan–Meier curves) of *Usp18*^*+/+*^ and *Usp18*^*−/−*^ mice (eight per group) injected intraperitoneally with VSV (1 × 10^8^ PFU per mouse), with log rank test [Mantel-Cox], with ****p < 0.0001. **h** Hematoxylin-and-eosin-stained images of lung sections from mice as in **e**. Scale bars, 50 µm.

To investigate the role of USP18 in the regulation of the viral infection, we measure the SeV viral protein using the SeV antibody in MEF cells after infection with SeV. The band with the strongest intensity corresponds to SeV P protein according to the molecular weight. Consistent with the function of USP18 in the regulation of IFN-β signaling, we observed an enhanced viral protein level of SeV in *Usp18*^*−/−*^ MEFs compared to that in *Usp18*^*+/+*^ MEFs (Fig. 3a). Furthermore, overexpression of USP18 in HEK293T cells resulted in an increased level of pIRF3 and correspondingly reduced level of SeV in a dosage-dependent manner (Fig. 3b). This phenomenon is not due to the transfection of plasmids since the overexpression of GFP in HEK293T cells could not affect the levels of pIRF3 and SeV (Fig. 3c).

To examine the physiological relevance of USP18 during RNA virus infection in vivo, we challenged *Usp18*^*+/+*^ and *Usp18*^*−/−*^ mice with VSV-GFP. To exclude the possibility that USP18 may affect the development of the immune system, we isolated the thymus, spleen, and peripheral lymph glands from *Usp18*^*+/+*^ and *Usp18*^*−/−*^ mice and observed that there was no substantial distinction between them (Supplementary Fig 3). We found, by ELISA analysis, that the production of IFN-β in the serum of *Usp18*^−/−^ mice was significantly lower than that of *Usp18*^*+/+*^ mice after infection with VSV (Fig. 3d). Moreover, we observed a more severe paralysis symptom in *Usp18*^−/−^ mice after infection, with a corresponding higher VSV titers in the brain (Fig. 3d). We also observed that VSV titers (Fig. 3e) and VSV-GFP protein (Fig. 3f) were significantly greater in the spleen, liver, and lung of *Usp18*^−/−^ mice than in those from *Usp18*^*+/+*^ mice. Furthermore, *Usp18*^−/−^ mice were less resistant to infection with VSV compared with *Usp18*^*+/+*^ mice through intraperitoneal injection (Fig. 3g), exhibiting significantly reduced survival time. Since the previous study has suggested that *Usp18*^−/−^ mice were more resistant to the VSV infection^31^, we also performed the intracerebral injection of VSV same as the previous study and observed that *Usp18*^−/−^ mice had a comparable survival time compared with *Usp18*^*+/+*^ mice (Supplementary Fig. 4). The difference in survival time is likely due to the different injection routes. Furthermore, hematoxylin-and-eosin staining showed greater infiltration of immune cells and injury in the lungs of *Usp18*^−/−^ mice, relative to that in the lungs of *Usp18*^*+/+*^ mice, after infection with VSV (Fig. 3h). These data demonstrated that *Usp18*^−/−^ mice were more susceptible than *Usp18*^*+/+*^ mice to infection with VSV with intraperitoneal injection, further confirming that USP18 plays an important role in the regulation of antiviral innate immunity against RNA virus infection.

### USP18 interacts with MAVS in the RLR signaling pathway

**Fig. 4:**
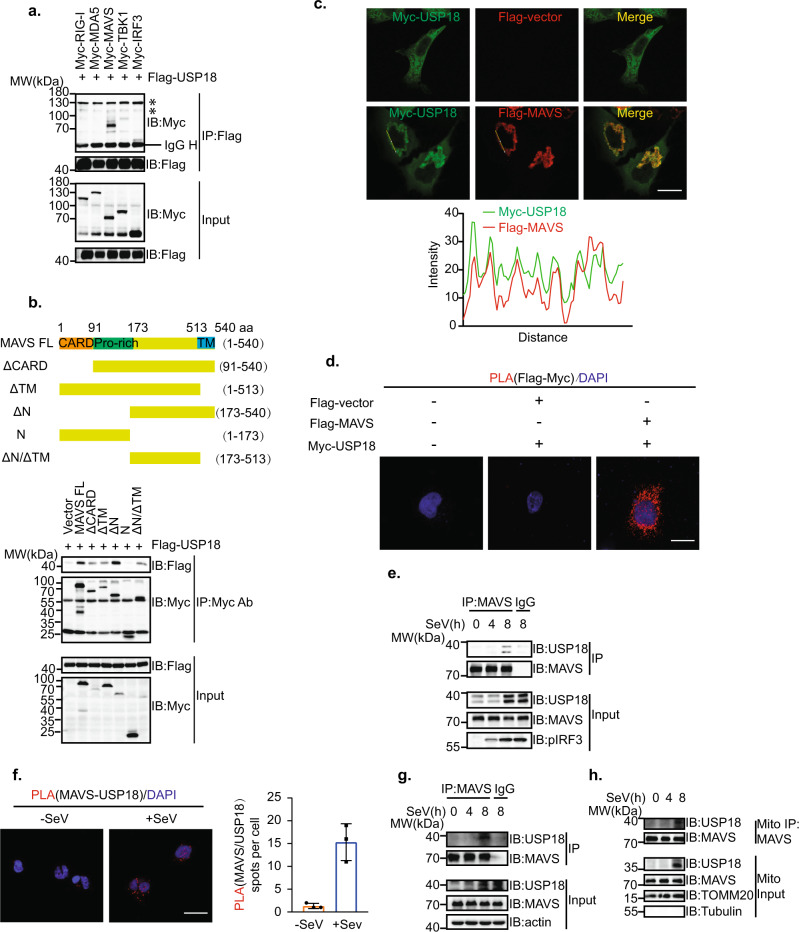
USP18 targets MAVS in the RLR signaling pathway. **a** Co-IP analysis of the interaction between USP18 and signaling molecules in HEK293T cells transfected with the plasmids expressing Flag-USP18 and Myc-RIG-I, MDA5, MAVS, TBK1, and IRF3. Asterisk represents unspecific signal. **b** Top panel: schematic representation of MAVS or MAVS truncations. Bottom panel: Co-IP analysis of the interaction between Flag-USP18 and Myc-MAVS or MAVS truncations in HEK293T cells. **c** Top panel: representative confocal images of immunofluorescence staining for Myc-USP18 only or Myc-USP18 colocalization with Flag-MAVS in HeLa cells, scale bar, 10 µm. Bottom panel: line profiling of Myc-USP18 with Flag-MAVS, and intensity of each line was quantified by ImageJ software and drawn by GraphPad Prism 8.0. **d** In situ PLA assay of the USP18-MAVS interaction in HeLa cells with indicated combinations using an anti-Flag and anti-Myc antibodies, scale bar, 10 µm. **e** Co-IP analysis of the interaction between USP18 and MAVS in THP-1 cells infected with SeV for 0–8 h, lysed and immunoprecipitated with a control immunoglobulin G (IgG), or anti-MAVS. **f** Left panel: In situ PLA assay of the USP18-MAVS interaction in THP-1 cells infected without or with SeV using anti-USP18 and anti-MAVS antibodies, scale bar, 10 µm. Right panel: quantification of interaction spots before and after SeV infection from 50 cells was drawn by GraphPad Prism 8.0. **g** Co-IP analysis of the interaction between USP18 and MAVS in RAW264.7 cells infected with SeV for 0–8 h. **h** Co-IP analysis of the interaction between USP18 and MAVS in the mitochondrial (Mito) fraction of THP-1 cells infected with SeV for 0–8 h.

Since USP18 is a mitochondria-associated protein and can increase MAVS ubiquitination, we hypothesized that USP18 can target MAVS to regulate antiviral innate immunity against RNA virus. To investigate whether USP18 specifically interacts with MAVS, we utilized the Co-IP assay to examine the interaction between the signaling molecules in the RLR signaling pathway and USP18. The results showed that USP18 mainly interacted with MAVS, while there was no or weak association with upstream molecules RIG-I or MDA5 and downstream molecules TBK1 or IRF3 in the RLRs signaling pathway (Fig. 4a). Furthermore, we found that USP18 interacted with the fragment of 173 aa to 573 aa in MAVS, located between the proline-rich and TM domain (Fig. 4b). Immunofluorescence assay showed that Myc-USP18 exhibited colocalization with Flag-MAVS (Fig. 4c). The spatial colocalization and interaction between USP18 and MAVS were further confirmed by in situ proximity ligation assay (PLA), in which the number of red spots displaying the interaction between USP18 and MAVS. There were no red spots when Myc-USP18 was co-transfected with the control Flag vector, while the transfection of both Myc-USP18 and Flag-MAVS resulted in significant numbers of red spots (Fig. 4d). Notably, we also observed that the endogenous interaction between USP18 and MAVS was enhanced following the SeV infection in THP-1 cells (Fig. 4e), indicating that USP18 may play a role in modulating the activity of MAVS. This enhanced complex formation of USP18 and MAVS was further validated through the PLA assay, in which the number of spots representing the endogenous USP18-MAVS complex increased significantly following SeV infection (Fig. 4f). The elevated endogenous interaction between MAVS and USP18 was also confirmed in the RAW264.7 cells upon SeV infection (Fig. 4g). Since MAVS is localized in mitochondria, we further isolated the mitochondrial fraction and observed an enhanced interaction between MAVS and USP18 in mitochondria following SeV infection (Fig. 4h). Together, these results suggested that USP18 primarily targets MAVS in the RLR signaling pathway.

### USP18 enhances the K63-linked polyubiquitination and aggregation of MAVS

**Fig. 5:**
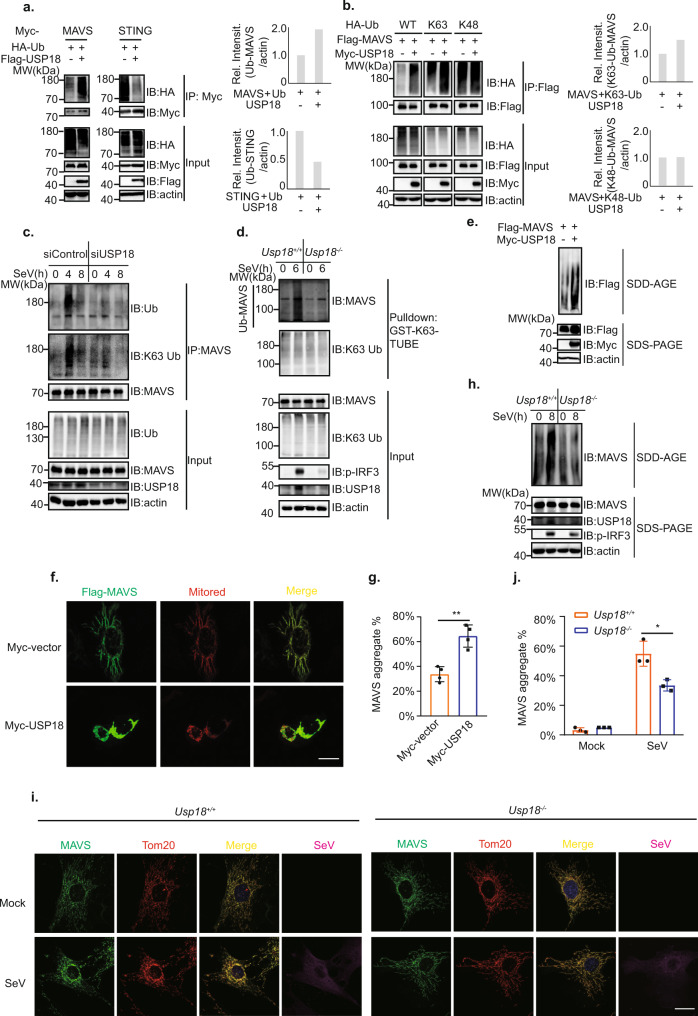
USP18 promotes the K63-linked polyubiquitination and subsequent aggregation of MAVS. **a** Immunoprecipitation analysis of ubiquitination of signaling molecules in HEK293T cells transfected with the plasmids expressing Myc-MAVS or STING, HA-Ub, and Flag vector or Flag-USP18. **b** Immunoprecipitation analysis of ubiquitination of MAVS in HEK293T cells transfected with the plasmids expressing Flag-MAVS, HA-wild-type, K63 only, or K48 only Ub, and Myc vector or Myc-USP18. **c** Immunoblot analysis of ubiquitination of endogenous MAVS in RAW264.7 cells transfected with siControl or siUSP18 followed by infection with SeV for indicated time points. **d** K63-Ub-TUBE analysis of ubiquitination of endogenous MAVS in *Usp18*^*+/+*^ and *Usp18*^*−/−*^ MEFs infected with SeV and harvested at indicated time points. **e** SDD-AGE (top) and SDS-PAGE (bottom) immunoblot analysis of MAVS aggregation in HEK293T cells transfected with the plasmids expressing Flag-MAVS with Myc vector or Myc-USP18. **f** Representative confocal images of immunofluorescence staining for HeLa cells transfected with the plasmids expressing Flag-MAVS, DsRED2-Mito together with Myc vector or Myc-USP18, scale bar, 10 µm. **g** Quantification of MAVS aggregate in HeLa cells transfected with the plasmids expressing Flag-MAVS, DsRED2-Mito together with Myc vector or Myc-USP18 (50 cells per sample). Data are presented with mean ± SD, with **p = 0.0019 (two-tailed Student’s *t* test). **h** SDD-AGE (top) and SDS-PAGE (bottom) immunoblot analysis of MAVS aggregation in *Usp18*^*+/+*^ and *Usp18*^*−/−*^ MEFs infected with SeV and harvested at indicated time points. **i** Representative confocal images of immunofluorescence staining for *Usp18*^*+/+*^ and *Usp18*^*−/−*^ MEFs harvested before infection or infection with SeV for 8 h, scale bar, 10 µm. **j** Quantification of MAVS aggregate in *Usp18*^*+/+*^ and *Usp18*^*−/−*^ MEFs harvested before infection or infection with SeV for 8 h (30 cells per sample). Data are presented with mean ± SD, with *p = 0.0328 (two-tailed Student’s *t* test).

Next, we investigated whether USP18 specifically modulates the ubiquitination level of MAVS, we transfected plasmids encoding USP18, ubiquitin together with signaling molecules in the RLRs pathway into HEK293T cells. We observed that USP18 only enhanced the ubiquitination level of MAVS rather than RIG-I, MDA5, and TBK1 (Fig. 5a and Supplementary Fig 5a), suggesting its specific regulation of MAVS ubiquitination in the RLR pathway. Consistent with a previous study^25^, our results showed that USP18 overexpression led to a decrease in the ubiquitination of STING (Fig. 5a). Since different ubiquitin linkages are related to distinctive functions, we next examined the type of ubiquitin linkage in MAVS that was promoted by USP18. Through transfecting K48- or K63-specific linkage Ub mutant, we observed that USP18 enhanced the K63-linked, instead of K48-linked, polyubiquitination of MAVS (Fig. 5b). To further exclude the possibility that USP18 may play a role in regulating the K48-linked polyubiquitination of MAVS, we examined the stability of MAVS. Overexpression of USP18 in HEK293T cells did not significantly affect the protein level of endogenous MAVS (Supplementary Fig 5b). Besides, knockout of USP18 in mouse embryonic fibroblast (MEF) cells did not affect the MAVS protein level with the infection of SeV (Supplementary Fig. 5c). Importantly, the turnover rate of MAVS was comparable between *Usp18*^*+/+*^ and *Usp18*^*−/−*^ MEFs under the treatment of Cycloheximide (CHX; Supplementary Fig. 5d), indicating USP18 could not affect K48-linked ubiquitination and protein degradation of MAVS. Next, we analyzed the effect of USP18 on the ubiquitination of endogenous MAVS through both immunoprecipitation and tandem ubiquitination binding entity (TUBE) assays. Consistent with the results from overexpression of USP18, siRNA knockdown of USP18 in RAW264.7 cells resulted in a decrease of K63-linked ubiquitinated MAVS (Fig. 5c). Since IP assay likely precipitates the ubiquitination proteins that interact with MAVS, we also utilized the TUBE assay with GST-K63-TUBE (Fig. 5d), which enrich the K63-linked ubiquitinated proteins^32,33^. We observed that MAVS of *Usp18*^−/−^ MEF displayed significantly less K63-linked polyubiquitination following viral infection compared with that of *Usp18*^+/+^ MEFs (Fig. 5d). Congruent with a previous study^25^, the depletion of USP18 in RAW264.7 cells or deletion of USP18 in MEFs led to an increase of K48-linked polyubiquitinated STING (Supplementary Fig. 6a, b).

As aforementioned, K63-linked polyubiquitination of MAVS promotes its aggregation, which is a prerequisite for the activation of downstream signaling. Given that USP18 enhances the K63-linked polyubiquitination of MAVS, we next examined whether USP18 can facilitate the aggregation of MAVS. Semi-Denaturating Detergent Agarose Gel Electrophoresis (SDD-AGE) analysis showed that overexpression of USP18 resulted in more formation of MAVS aggregates compared with the control Myc vector (Fig. 5e). Furthermore, we observed that the transfection of USP18 together with MAVS in HeLa cells led to more MAVS aggregate, exhibiting decreased mitochondrial skeleton length and cytosol area (Fig. 5f, g). Consistent with the observation from overexpression of USP18, *Usp18*^*−/−*^ MEF displayed decreased aggregation of MAVS demonstrated by both SDD-AGE and immunofluorescence assays compared with *Usp18*^*+/+*^ MEFs upon SeV infection (Fig. 5h–j). Altogether, these data demonstrated that USP18 promotes K63-linked ubiquitination and aggregation of MAVS during RNA virus infection.

### USP18 modulates the ubiquitination of MAVS independent of its enzymatic activity

**Fig. 6:**
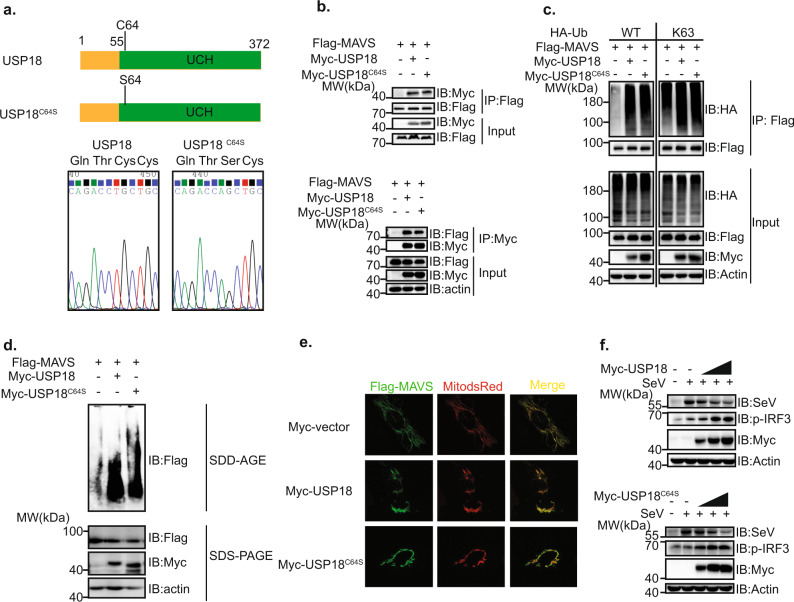
Enzymatic activity of USP18 is dispensable for its function in RLR pathway. **a** Top panel: schematic diagram of USP18 and USP18 with C64S mutation (USP18^C64S^). Bottom panel: DNA sequencing result of plasmids expressing Myc-USP18 or USP18^C64S^. **b** Co-IP analysis of the interaction between MAVS and USP18 or USP18^C64S^ in HEK293T cells transfected with the plasmids expressing Flag-MAVS, with Myc vector, Myc-USP18 or Myc-USP18^C64S^ utilizing either anti-Flag magnetic beads (top panel) or anti-Myc magnetic beads (bottom panel). **c** Immunoprecipitation analysis of ubiquitination of MAVS in HEK293T cells transfected with the plasmids expressing Flag-MAVS, HA-wild type or HA-K63 only Ub, and Myc vector, Myc-USP18, or Myc-USP18^C64S^. **d** SDD-AGE (top) and SDS-PAGE (bottom) analysis of MAVS aggregation in HEK293T cells transfected with the plasmids expressing Flag-MAVS, with Myc vector, Myc-USP18, or Myc-USP18^C64S^. **e** Representative confocal images of immunofluorescence staining for HeLa cells transfected with the plasmids expressing Flag-tagged MAVS, DsRED2-Mito together with Myc vector, Myc-USP18, or Myc-USP18^C64S^, scale bar, 10 µm. **f** Immunoblot analysis of SeV and pIRF3 in lysates of HEK293T cells transfected with Myc-USP18 (top) and Myc-USP18^C64S^ (bottom) in different dosages (1 µg, 2 µg, and 3 µg) for 12 h followed by infection with SeV for 8 h.

Given that USP18 is a DUB, but can efficiently enhance the polyubiquitination level of MAVS, we propose two possible scenarios. First, USP18 can stabilize and increase the protein level of the E3 ubiquitin ligase for MAVS dependent on its enzymatic activity. Second, USP18 serves as an adaptor protein to recruit the E3 ubiquitin ligase for MAVS rather than relying on its enzymatic activity. To test the first scenario, we mutated the cysteine residue 64 of USP18 to serine (USP18^C64S^; Fig. 6a), which lost the enzymatic activity from previous study^26^. We observed that the interaction between MAVS and USP18 was not affected by this mutation (Fig. 6b). Furthermore, we observed that USP18^C64S^ could enhance the K63-linked polyubiquitination of MAVS like wild-type USP18 (Fig. 6c). Consistently, USP18^C64S^ resulted in the aggregation of MAVS to the extent comparable to wild-type USP18 through both SDD-AGE and immunofluorescence assays (Fig. 6d, e). We also found that transfection of both USP18 and USP18^C64S^ into HEK293T cells led to an increase of phosphorylated IRF3 and subsequently a decrease of SeV protein in a dosage-dependent manner (Fig. 6f). In conclusion, these data in combination indicated that USP18 enhances the K63-linked polyubiquitination of MAVS and the antiviral innate immune response irrespective of its enzymatic activity.

### USP18 promotes MAVS K63-linked polyubiquitination dependent on E3 ligase TRIM31

**Fig. 7:**
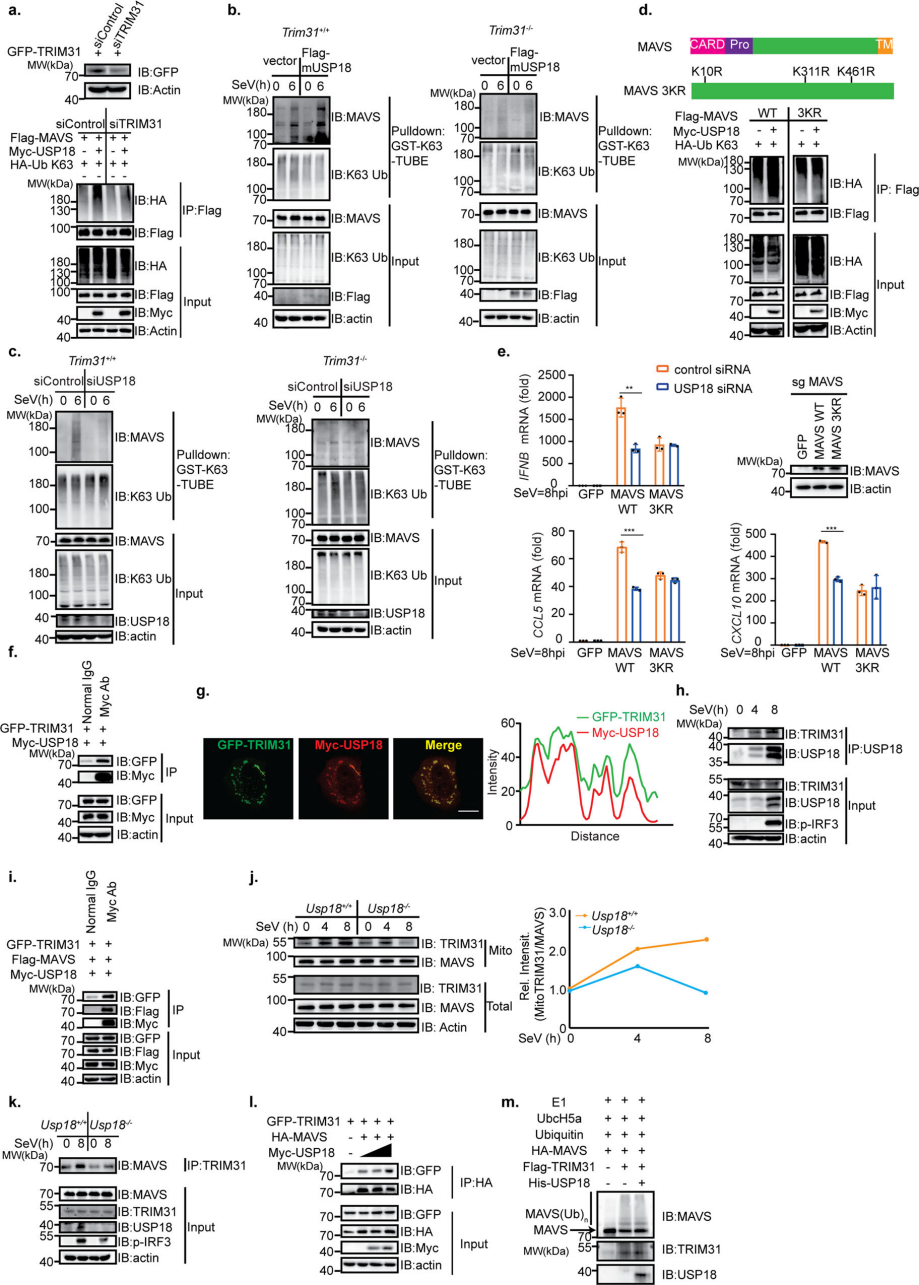
USP18 interacts with TRIM31 to promote the K63-linked polyubiquitination of MAVS. **a** Top panel: immunoblot analysis of TRIM31 and actin in HEK293T cells transfected with control siRNA (siControl) or siRNA targeting TRIM31 (siTRIM31) for 12 h followed by transfection of plasmids encoding GFP-TRIM31 for 24 h. Bottom panel: immunoprecipitation analysis of ubiquitination of MAVS in HEK293T cells transfected with siControl and siTRIM31 followed by transfection of the plasmids expressing Flag-MAVS, HA-K63 only Ub, and Myc vector, Myc-USP18. **b** K63-TUBE analysis of ubiquitination of endogenous MAVS in *Trim31*^*+/+*^ and *Trim31*^*−/−*^ MEFs transfected with pLenti vector or pLenti-Flag mUSP18 followed by SeV infection for indicated time points. **c** Left panel, K63-TUBE analysis of ubiquitination of endogenous MAVS in RAW264.7 cells transfected with siControl or siUSP18 followed by infection with SeV for indicated time points. Right panel, K63-TUBE analysis of ubiquitination of endogenous MAVS in *Trim31*^*−/−*^ MEFs transfected with control siRNA or USP18 siRNA followed by SeV infection for indicated time points. **d** Top panel: schematic diagram of wild-type MAVS and MAVS 3KR mutant (K11, 311, and 461R). Bottom panel: immunoprecipitation analysis of ubiquitination of wild-type-MAVS or MAVS 3KR mutant in HEK293T cells transfected with plasmids expressing Flag-MAVS or Flag-MAVS 3KR, HA-K63 only Ub, and Myc vector or Myc-USP18. **e** qRT-PCR analysis of *IFNB*, *CCL5*, and *CXCL10* expression level and immunoblot analysis of MAVS in MAVS KO HeLa cells transfected with control siRNA or USP18 siRNA and reconstituted with GFP, WT MAVS, or MAVS 3KR plasmid followed by infection with SeV for 8 h. Representative data were collected and expressed as mean ± SD from three independent experiments, with **p = 0.0023 (*IFNB*), ***p < 0.001 (*CCL5*), ***p < 0.001 (*CXCL10*) (two-tailed Student’s *t* test). **f** Co-IP analysis of the interaction between TRIM31 and USP18 in HEK293T cells transfected with GFP-TRIM31 and Myc-USP18. **g** Representative confocal images of immunofluorescence staining for Myc-USP18 colocalization with GFP-TRIM31 in HeLa cells, scale bar, 10 µm. Right panel: line profiling of Myc-USP18 with Flag-MAVS, and intensity of each line was quantified by ImageJ software. **h** Co-IP analysis of the endogenous interaction between TRIM31 and USP18 in THP-1 cells infected with SeV for indicated time points. **i** Co-IP analysis of interaction between USP18, TRIM31, and MAVS in HEK293T cells transfected with Flag-MAVS, Myc-USP18, and GFP-TRIM31. **j** Immunoblot analysis of the indicated proteins after isolating the mitochondrial fraction of the MEFs following SeV infection for indicated time points. **k** Co-IP analysis of interaction between endogenous TRIM31 and MAVS in *Usp18*^*+/+*^ and *Usp18*^*−/−*^ MEFs infected with SeV for 0–8 h and harvested at indicated times. **l** Co-IP analysis of interaction between TRIM31 and MAVS in HEK293T cells transfected with HA-MAVS, GFP vector or GFP-TRIM31, and different dosages of Myc-USP18. **m** In vitro ubiquitination assay of MAVS in the presence or absence of USP18.

Since USP18 promoted the polyubiquitination of MAVS independent of its enzymatic activity, we hypothesized that USP18 likely recruits an E3 ligase for K63-linked polyubiquitination of MAVS. TRIM31 is the only identified E3 ligases catalyzing the K63-linked polyubiquitination of MAVS. Therefore, we investigated whether the effect of USP18 on the ubiquitination of MAVS was dependent on TRIM31. We observed that knockdown of TRIM31 in HEK293T cells led to the abrogation of the enhanced K63-linked ubiquitination of MAVS by USP18 (Fig. 7a). Furthermore, overexpression of USP18 in *Trim31*^*+/+*^ MEFs, rather than *Trim31*^*−/−*^ MEFs, led to an enhanced K63-linked polyubiquitination of MAVS (Fig. 7b). To further delineate the relationship between USP18 and TRIM31 in regulating the ubiquitination of MAVS, we knocked down USP18 in the presence or absence of TRIM31. We observed that ablation of USP18 in the presence of TRIM31 could decrease the K63-linked polyubiquitination level of MAVS, while knockdown of USP18 in absence of TRIM31 could not further downregulate the ubiquitination level of MAVS (Fig. 7c), suggesting that function of USP18 in mediating the K63-linked polyubiquitination of MAVS is upstream of and dependent on TRIM31. The previous study from our lab has identified that TRIM31 catalyzes the K63-linked polyubiquitination at lysine residues 11, 311, and 461 of MAVS. Therefore, USP18 should not enhance the K63-linked polyubiquitination of MAVS with K11R, K311R, and K461R if its effect on MAVS is dependent on TRIM31. As expected, we found that K63-linked polyubiquitination of MAVS K11R, K311R, and K461R cannot be enhanced by USP18 compared with WT MAVS (Fig. 7d). Next, we examined whether USP18 still regulates SeV-induced type I response with MAVS 3KR. To test this, we first constructed the MAVS KO HeLa cells (Supplementary Fig 7a). Furthermore, we found that knockdown of USP18 could lead to a decrease of *IFNB*, *CCL5*, and *CXCL10* in MAVS KO HeLa reconstituted with WT MAVS instead of MAVS 3KR following SeV infection (Fig. 7e), indicating that USP18 regulates the RLR-mediated type-I interferon response through regulating the TRIM31-catalyzed ubiquitination of MAVS.

### USP18 promotes the interaction between MAVS and TRIM31 in mitochondria

Next, we investigated the mechanism USP18 promoted the ubiquitination of MAVS through TRIM31. Since the effect of USP18 was independent of its enzymatic activity, the stability of TRIM31 should not be affected by USP18. As we expected, the overexpression of USP18 in HEK293T cells did not affect the protein level of TRIM31 (Supplementary Fig 7b). Therefore, we hypothesized that USP18 interacts with TRIM31 and subsequently enhances the interaction between TRIM31 and MAVS. To test our hypothesis, we first examined whether the interaction between USP18 and TRIM31 existed. We observed that USP18 did interact with TRIM31 through the Co-IP assay (Fig. 7f). Immunofluorescence assay showed that USP18 can colocalize with TRIM31 (Fig. 7g), further suggesting the interaction between USP18 and TRIM31. More importantly, we observed that the interaction between endogenous USP18 and TRIM31 was increased following SeV infection (Fig. 7h), indicating USP18 may control the activity of TRIM31. Moreover, we observed that USP18, TRIM31, and MAVS form a complex through Co-IP assay, suggesting that USP18 likely serves as the scaffold protein between MAVS and TRIM31 (Fig. 7i). TRIM31 was previously reported to translocate to mitochondria to interact with MAVS following viral infection. However, the mechanism regulating this process is not completely understood. Therefore, we hypothesize that USP18 recruits TRIM31 to mitochondria to facilitate the interaction between TRIM31 and MAVS. Consistent with the previous study^13^, we observed that TRIM31 was efficiently enriched in mitochondria in *Usp18*^*+/+*^ MEFs following SeV infection. However, this enrichment was significantly impaired in *Usp18*^*−/−*^ MEF, suggesting that USP18 was crucial for the translocation of TRIM31 to mitochondria (Fig. 7j). As a control, the MAVS level in mitochondrial fraction was not affected in the presence or absence of USP18 (Fig. 7j), suggesting a specific regulation of TRIM31 localization by USP18. Given that USP18 was critical for TRIM31 localization to mitochondria, we speculated USP18 should modulate the interaction between MAVS and TRIM31. We observed that the interaction between MAVS and TRIM31 following viral infection was significantly impaired in *Usp18*^*−/−*^ MEFs compared with *Usp18*^*+/+*^ MEFs (Fig. 7k). Furthermore, overexpression of USP18 could enhance the interaction between MAVS and TRIM31 in a dosage-dependent manner (Fig. 7l). Interestingly, we also observed that the addition of USP18 in the in vitro ubiquitination assay did not enhance the ubiquitination of MAVS catalyzed by TRIM31 different from the ubiquitination assay within cells (Fig. 7m) further indicating that USP18 very likely bridges the link between TRIM31 and MAVS in vivo. Taken together, these data suggested that USP18 serves as a scaffold to recruit TRIM31 into mitochondria to promote MAVS ubiquitination and aggregation to regulate anti-viral innate immunity.

## Discussion

Ubiquitination plays an indispensable role in regulating the activity of MAVS^11^. Ubiquitination is a reversible process balanced by both E3 ligases and DUBs. Previous studies have reported numbers of E3 ligases in regulating the MAVS activation and stability, but limited DUBs were reported to modulate the ubiquitination level of MAVS. Given the specific localization of MAVS, we intended to identify a mitochondrial DUB in regulating the ubiquitination level and subsequent activity of MAVS. Interestingly, we observed that the USP18, within the DUBs likely present in mitochondria, significantly promoted the polyubiquitination of MAVS in our screening system. Besides, we found that USP18 is partially localized in the mitochondrial fraction. More importantly, USP18 is enriched in the mitochondrial fraction following the viral infection. Since different ubiquitin chain linkages exert distinctive functions, we next investigated the type of polyubiquitination mediated by USP18. We found that USP18 promoted the K63-linked rather than K48-linked polyubiquitination of MAVS, which was supported by several scenarios. First, overexpression of ubiquitin linkage-specific mutants and USP18 resulted in the enhanced K63-linked instead of K48-linked polyubiquitination. Second, the stability of MAVS was not changed in the presence or absence of USP18, which excluded the possibility that USP18 affects the K48-linked polyubiquitination of MAVS since K48-linked ubiquitin chains signal for protein degradation. Third, immunoprecipitation followed by probing with K63-Ub specific antibody or TUBE assay against K63-specific linkages further strengthened the observation that deficiency of USP18 led to the decrease of K63-linked polyubiquitination of MAVS during viral infection. Last, we investigated whether USP18 can facilitate the aggregation of MAVS, given K63-linked polyubiquitination promotes the aggregation of MAVS. As expected, overexpression of USP18 caused the formation of more MAVS aggregates, while the deletion of USP18 negatively regulated the aggregation of MAVS upon viral infection. These results explore a novel function of USP18 in regulating the ubiquitination and aggregation of MAVS.

The aggregation of MAVS is critical for the viral-induced type-I interferon signaling activation^4^, and USP18 regulates the aggregation of MAVS. Therefore, we next examined whether the deletion of USP18 also affects the downstream events. Upon SeV or EMCV infection, macrophages or MEFs with the deficiency of USP18 showed impaired phosphorylation of TBK1 and IRF3, expression of *Ifnb* and *Ccl5*, and production of IFN-β, indicating that USP18 plays a positive role in regulating viral-induced type-I interferon response. Consistently, *Usp18*^*−/−*^ mice were more susceptible to viral infection compared with wild-type mice. Previously, USP18 is identified as a negative regulator of type-I interferon signaling via inhibiting the interaction between JAK and IFNAR. Consequently, USP18 deficiency enhances viral resistance after type I IFN stimulation. However, a recent study suggests that USP18 cooperates with USP20 to stabilize STING and promotes the antiviral type-I interferon response^25^. Therefore, the macrophages with the deletion of USP18 result in more production of HSV-1 viral particles. In our study, we found that USP18 facilitates the type-I interferon response upon SeV or EMCV infection through promoting the K63-linked polyubiquitination and aggregation of MAVS. Moreover, two recent studies have suggested that the negative regulation of USP18 on type-I interferon signaling is to prevent the overwhelmed immune response and auto-immune disease. Two studies observe that humans or mice with the deletion of USP18 are prone to have type 1 interferonopathy, leading to severe pseudo-TORCH syndrome or destructive brain interferonopathy^34,35^. Recently, a clinical study further underlies the supreme importance of USP18 in regulating immune homeostasis^36^. USP18 deficiency in neonate leads to a severe pseudo-TORCH syndrome, showing manifestation such as perinatal-onset intracranial hemorrhage, calcifications, brain malformations without detectable pathogen. The severe clinical outcome caused by loss of function USP18 mutant can be rescued by JAK inhibitor ruxolitinib, suggesting that the clinical manifestation caused by loss of USP18 can be reversed through interfering with the JAK-STAT pathway. This study again emphasizes the importance of USP18 in negatively regulating the downstream effect of IFN or inflammatory cytokines. But our study and studies from other labs have found that USP18 deficiency in mice led to an enhanced virus load in the brain and a slower clearing of both RNA and DNA viruses, such as VSV and HSV-1^25,37^. Moreover, the same study about USP18 stabilization of STING also found that USP18 deficiency led to an impaired interferon production upon the RNA virus infection, again indicating that USP18 is an important positive regulator of innate immune signaling against viral infection^25^. Another study also found USP18 overexpression promoted the nuclear translocation of p65 and p50 to restrict the PRRSV replication^38^. Overall, our study and other studies indicate USP18 plays different roles in different steps of type-I interferon signaling, facilitating the viral-induced immune response but dampening the interferon-induced overwhelmed response.

It is an intriguing observation that USP18, as a DUB, enhanced the polyubiquitination of MAVS. USP18^C64S^, the mutation of cysteine residues in the enzymatic center of USP18, can interact with MAVS and enhances the ubiquitination as well as aggregation of MAVS comparable to the wild-type USP18, suggesting USP18 is involved in MAVS-mediated signaling pathway independent of its enzymatic activity. Furthermore, we found that the effect of USP18 on MAVS was dependent on the E3 ligase TRIM31 through a series of experiments. First, USP18 cannot enhance the polyubiquitination of MAVS with the knockdown or knockout of TRIM31. Second, USP18 cannot facilitate the polyubiquitination of MAVS 3KR, which cannot be ubiquitinated by TRIM31. TRIM31 was previously reported to be recruited to mitochondria following RNA virus infection, promoting the K63-linked polyubiquitination and subsequent aggregation of MAVS^13^. However, the mechanism regulating the re-localization of TRIM31 to mitochondria and interaction between TRIM31 and MAVS is unknown yet. Therefore, we speculate that USP18 may act as an adaptor protein to connect TRIM31 and MAVS. Studies from other labs have found that O-GlcNAcylation of MAVS by OGT promotes its interaction with TRIM31 and subsequent K63-linked polyubiquitination^39,40^, while RAC1 and FAF1 negatively regulate their interaction through distinctive mechanisms^41,42^. In our study, we found that USP18 serves as a critical adaptor protein and facilitates the re-localization of TRIM31 and interaction between TRIM31 and MAVS. The deletion mapping found that USP18 interacted with residues of 171–513 of MAVS, and the previous study suggested that TRIM31 bound with the proline-rich domain of MAVS. The fact that different domains of MAVS mediate its interaction with TRIM31 and USP18 further suggests that USP18 is very likely to promote the interaction between TRIM31 and MAVS. Different from USP18, RAC1 also interacts with the proline-rich domain of MAVS, likely competing with TRIM31 for interaction with MAVS. The domains of USP18 in governing the interaction with TRIM31 and MAVS warrants further investigation. Besides, how these proteins and post-translational modifications cooperate to modulate the activity of MAVS spatially and temporally should be carefully dissected and investigated.

In summary, we have identified a deubiquitinase USP18 as a host factor modulating MAVS aggregation during the infection of RNA viruses. Following viral infection, USP18 is enriched in mitochondria and promotes the mitochondrial translocation of TRIM31 from the cytoplasm and interaction between TRIM31 and MAVS, consequently enhancing TRIM31-mediated K63-linked polyubiquitination and subsequent aggregation of MAVS. Our study suggests a complicated regulatory mechanism of MAVS aggregation, emphasizing its supreme importance in the innate antiviral response.

## Methods

### Cell culture

Human HEK293T, HeLa, THP-1, and mouse RAW264.7 cells were purchased from American Type Culture Collection. THP-1 cells were cultured in RPMI-1640 supplemented with 10% FBS, 100 U/ml penicillin, and 100 μg/ml streptomycin. The rest of the cells were cultured in DMEM supplemented with 10% FBS, 100 U/ml penicillin, and 100 μg/ml streptomycin. Mouse embryonic fibroblasts (MEFs) from *Usp18*^*+/+*^ and *Usp18*^*−/−*^ mice were prepared from day 15 embryos, and peritoneal macrophages (PMs) were harvested from mice 4 days after thioglycollate (BD) injection. Both MEFs and PMs were cultured in DMEM supplemented with 10% FBS, 100 U/ml penicillin, and 100 μg/ml streptomycin.

### Mice

*Usp18*^*+/−*^ mice were obtained from Prof. Bo Zhong (Wuhan University, China), which were originally established by Prof. Dong-er Zhang (University of California at San Diego)^43^. *Usp18*^+/−^ mice were a mixed Sv129-C57BL/6 background. *Usp18*^*+/−*^ mice were crossed to obtain *Usp18*^*+/+*^ and *Usp18*^*−/−*^ mice. All mice were housed in the specific pathogen-free animal facility with 40–70% humidity and daily cycles of 12 h of light at 23 °C and 12 h of dark at 21 °C at Shandong University and all animal experiments were under protocols approved by the Institutional Animal Care and Use Committee of Shandong University. In all, 8–12-weeks-old littermate male mice were used for the experiments.

### Viruses

Sendai virus (SeV) was obtained from the China Center for Type Culture Collection (Wuhan University, China). Vesicular Stomatitis Virus with GFP insertion (VSV-GFP) and Encephalomyocarditis virus (EMCV) were kindly provided by Dr. Hong Meng (Institute of Basic Medicine, Shandong Academy of Medical Sciences, China) and Dr. Yingli Shang (Shandong Agricultural University), respectively.

### Plasmid constructs and small interfering RNA

The plasmid expressing Myc-USP18 of *Homo sapiens* was kindly provided by Dr. Li Bin (Shanghai Institute of Immunology). *USP18* cDNA of *Mus musculus* was amplified from peritoneal macrophages by standard PCR and cloned into pLVX-IRES-Puro plasmids. Plasmids encoding the full-length MAVS or MAVS truncations, TRIM31, HA-ubiquitin, or ubiquitin mutants have been described previously^13^. HA tagged MAVS and Flag-tagged TRIM31 were PCR amplified to replace the DHFR coding sequence of DHFR control plasmid (NEB). Deletion, truncation, and point mutations were generated by the QuikChange site-directed mutagenesis kits according to the manufacturer’s manual (Toyobo, SMK-101). pDsRED2-Mito was kindly provided by Dr. Jian Li (Shandong University). All constructs were confirmed by DNA sequencing. For transient transfection of plasmids into HEK293T and HeLa cells, Lipofectamine 2000 (Invitrogen) reagent was used.

The small interfering sequences were as follows: human *USP18*: 5′- CUGGUUGGUUUACACAACATT-3′, mouse *Usp18*: 5′-GCUGCUCAACUCUACCUUATT-3′, human *TRIM31*: 5′-GGACCACAAAUCCCAUAAUTT-3′, and Scrambled control sequences: 5′-UUCUCCGAACGUGUCACGUTT-3′. All these siRNAs were obtained from GenePharma. For transient transfection of siRNA duplexes into HEK293T, THP-1, RAW264.7, and peritoneal macrophage cells, Lipofectamine RNAiMAX (Invitrogen) reagent was used according to the manufacturer’s manual.

### Reagents and antibodies

HSV-60 is a 60-bp oligonucleotide containing viral DNA motifs derived from the HSV-1 genome. Poly(I:C)-LMW and Poly(I:C)-HMW were purchased from InvivoGen. E1, UbcH5a, and ubiquitin (WT) were purchased from Boston Biochem. Recombinant Mouse IFN-β was purchased from R&D systems. Anti-mouse IFNAR-1 antibody (MAR1-5A3) was purchased from Biolegend. Rabbit anti-HA Ab (600-401-384) was from Rockland; Rabbit anti-USP18 (D4E7), rabbit anti-GFP (D5.1) XP, rabbit anti-DYKDDDDK Tag (D6W5B), mouse anti-ubiquitin (P4D1), rabbit anti-K48 linkage-specific ubiquitin (D9D5), rabbit anti-K63 linkage-specific ubiquitin (D7A11), rabbit anti-IRF3 (D83B9), rabbit anti-pIRF3 (4D46), rabbit anti-TBK1 (3031S), rabbit anti-pTBK1 (D52C2), and normal rabbit IgG Abs were from Cell Signaling Technology. These antibodies were all used at 1:1000 dilution for western blotting analysis; Mouse anti-MAVS, mouse anti-α tubulin, and normal mouse IgG Abs were from Santa Cruz Biotechnology. These antibodies were all used at 1:1000 dilution for western blotting analysis, while MAVS was used at 1:50 dilution for immunofluorescence analysis; Mouse anti-actin, rabbit-anti-STING, and rabbit-anti-human TRIM31 Abs were from proteintech. Actin antibody was used at 1:5000 dilution, while STING and TRIM31 antibodies were used at 1:1000 dilution; Mouse anti-Flag M2 and rabbit anti-mouse TRIM31 Abs were from Sigma Aldrich; Mouse anti-Myc (9E10) Ab was from Origene; Rabbit anti-TOM20 Ab and rabbit anti-USP18 Ab was from Abclonal; Rabbit anti-Sendai virus Ab for Western Blotting was from MBL; Chicken anti-Sendai virus antibody (ab33988) for immunofluorescence was from Abcam. These antibodies were all used at 1:1000 dilutions for western blotting analysis.

### RNA purification and qRT-PCR

Total RNA was extracted with an RNA extraction kit (Fastagen Biotech Co., RNAfast200) and reversed-transcribed with PrimeScript RT-PCR kit (Takara RR014B) following the manufacturer’s instructions. Real-time PCR was performed with the SYBR Green PCR Master Mix (Roche) on ABI 7300 Detection System (Applied Biosystems). The primers for qPCR analysis were listed in the Supplementary Table 1. Data were normalized by the level of β–actin expression in each sample. 2^−△△Ct^ method was used to calculate relative expression changes. The sequences of the primers for qPCR analysis were listed in the supplementary primer table.

### Enzyme-linked immunosorbent assay

ELISA Kits (Bio legend, 439407) was utilized to measure the concentrations of IFN-β in culture supernatants and sera according to the manufacturer’s instructions.

### Co-immunoprecipitation assay

For Co-immunoprecipitation (Co-IP) assay, cells were collected 24 h after transfection and lysed in lysis buffer [1.0% (v/v) NP-40, 50 mM Tris-HCl, pH 7.4, 50 mM EDTA, 0.15 M NaCl] supplemented with a protease inhibitor cocktail (Sigma), and a phosphatase inhibitor cocktail (Sigma). After centrifugation for 10 min at 14,000 × *g*, supernatants were collected and incubated with the indicated antibodies followed by the addition of protein A/G beads (Santa Cruz), with Anti-Flag magnetic beads (Bimake), anti-Myc magnetic beads (Bimake), or anti-HA magnetic beads (Bimake). After incubation overnight at 4°, beads were washed four times with lysis buffer. Immunoprecipitates were eluted by boiling with 2×SDS loading buffer containing 100 mM Tris-HCl pH 6.8, 4% (w/v) SDS, 20% (v/v) glycerol, 0.2% (w/v) bromophenol blue, and 1% (v/v) 2-mercaptoethanol.

### Immunoblot analysis

For immunoblot analysis, cells or tissues were lysed with M-PER Protein Extraction Reagent (Pierce) supplemented with a protease inhibitor and a phosphatase cocktails (Sigma). Protein concentrations in the extracts were examined with a bicinchoninic acid assay (Pierce 23225) to make equal amount for different samples. Total cell lysates or immunoprecipitates prepared as described above were electrophoretically separated by SDS-PAGE, transferred onto a polyvinylidene difluoride membrane (Millipore), blocked with 3% (w/v) bovine serum albumin (BSA), probed with indicated primary antibodies and corresponding secondary antibodies, and visualized by ECL western blotting detection reagent (Pierce). Images were taken with SageCapture and processed with Adobe photoshop and Adobe Illustrator.

### Subcellular fractionation

The THP-1 cells infected with SeV or left uninfected for 6 h were washed with PBS and lysed by douncing in mitochondria isolation buffer (Beyotime Biotechnology). The homogenized cells were centrifuged at 1000 × *g* for 10 min at 4 °C to get rid of the nucleus. The supernatant was transferred and centrifuged at 3000 × *g* for 10 min at 4 °C to form a pellet, which was the mitochondria (P3). The supernatant was further centrifuged at 50,000 × *g* for 65 min to precipitate the ER (P50).

### Ubiquitination assay

For analysis of MAVS ubiquitination in HEK293T cells, HEK293T cells were transfected with the indicated plasmids, and then whole-cell extracts were immunoprecipitated with the corresponding antibody. For analysis of the ubiquitination of endogenous MAVS and STING in RAW264.7 cells, RAW264.7 cells were treated with the indicated stimulation, then whole-cell extracts were immunoprecipitated with anti-MAVS or anti-STING and analyzed by immunoblot with indicated antibodies.

### Purification of endogenous ubiquitin conjugates

Tandem ubiquitin-binding entities (TUBEs) were used to purify endogenous ubiquitin conjugates from RAW264.7 and MEF cell lysates. Glutathione-S-transferase (GST) fused to K63-Ub-TUBE were described as previously for isolation of K63-Ub chains^32,33^. RAW264.7 or MEF cells were lysed in ice-cold TUBE lysis buffer (50 mM Tris-HCl, pH 7.5, 0.15 M NaCl, 1 mM EDTA, 1% NP-40, 10% glycerol) supplemented with 50 µM PR-619 (Selleckchem), a protease inhibitor cocktail (Sigma), and a phosphatase inhibitor cocktail (Sigma). To purify ubiquitinated conjugates, GST-K63-Ub-TUBE was incubated with cell lysates and pulled down by GST beads (Qiagen). The GST beads were washed with TBS-T buffer (20 mM Tris-HCl, pH 8.0, 0.15 M NaCl, 0.1% Tween-20) for four times before boiling in 2× SDS loading buffer. The elutes were subject to SDS-PAGE electrophoresis and immunoblot analysis.

### In vitro ubiquitination assay

Flag-TRIM31 and HA-MAVS proteins were expressed with a TNT Quick Coupled Transcription/Translation System kit (Promega, L1171) according to the manufacture’s protocol. His-USP18 protein was bought from (Abiotech, Jinan, China), which was purified with nickel beads. In vitro ubiquitination assays were performed as described previously. Briefly, E1, UbcH5a, HA-MAVS, reaction buffer, ubiquitin, ATP, and MgCl_2_ were mixed with or without Flag-TRIM31 and His-USP18. The reaction mix was incubated at 30 °C for 1 h followed by addition of 6× SDS loading buffer at 95 °C for 10 min to stop the reaction.

### Native polyacrylamide gel electrophoresis

The IRF3 dimerization assay was performed as described previously^44^. Briefly, gels were pre-run with running buffer (25 mM Tris-HCl, pH 8.4, 192 mM glycine, in the presence or absence of 0.2% deoxycholate in the cathode and anode buffers, respectively) at 45 mA for 30 min. The lysate was centrifuged at 120,000 rpm for 10 min to remove the insoluble fraction. The sample was mixed with 2× loading buffer (125 mM Tris-Cl, pH 6.8, 30% glycerol, and 0.002% bromophenol blue) and was applied to the gel. The samples were electrophoresed at 25 mA for 50 min in a cold room for further analysis.

### Semi-denaturing detergent agarose gel electrophoresis

Semi-denaturing detergent agarose gel electrophoresis (SDD-AGE) analysis was performed as described previously^13^. In brief, harvested cells resuspended in mitochondrial isolation buffer (Beyotime Biotechnology) were subjected to dounce-homogenization to lyse the cell. The homogenate was centrifuged at 700 × *g* for 10 min at 4 °C to spin out the cell debris and nucleus. The supernatant was further centrifuged at 10,000 × *g* for 30 min at 4 °C to pellet the intact crude mitochondria. Crude mitochondria (P5) were lysed in 1×sample buffer (0.5× TBE, 10% glycerol, 2% SDS and 0.0025% bromophenol blue) and loaded onto a vertical 1.5% agarose gel (Bio-Rad). The proteins were transferred to an Immobilon membrane (Millipore) as mentioned above for further immunoblot analysis after electrophoresis in the running buffer (0.5× TBE and 0.1% SDS) for 35 min with a constant voltage of 100 V at 4 °C.

### Confocal microscopy

HeLa or primary *Usp18*^*+/+*^ and *Usp18*^*−/−*^ MEFs were grown on 12-well slides 1 day before transfection or infection. HeLa cells were transfected with indicated plasmids (each plasmid 0.5 µg), and MEFs were mock-infected or infected with SeV. Cells were then fixed in 4% paraformaldehyde, permeabilized with 0.2% Triton X-100, and blocked with phosphate-buffered saline (PBS) containing 5% horse serum and 1% BSA. The fixation, permeabilization, and blocking buffer were all purchased from Beyotime Biotechnology. The cells were then reacted with indicated primary antibodies at 4 °C overnight, rinsed, and reacted with corresponding secondary antibodies (Invitrogen). Nuclei were counterstained with DAPI (Abcam). Images were taken with a Zeiss LSM780 confocal microscope and processed with Zeiss Zen software.

### Proximity ligation assay

HeLa or THP-1 cells were fixed and permeabilized as mentioned above. Proximity ligation assay (PLA) was performed according to the manufacturer’s instructions (Duolink, Sigma-Aldrich, DUO92101), and nuclei were counterstained with DAPI (Abcam). Fluorescent red spots were detected by using a Zeiss LSM780 confocal microscope. The interaction red spots per cell were quantified using the ImageJ software.

### Viral infection in vivo

For in vivo viral infection studies, eight-week-old *Usp18*^*+/+*^ and *Usp18*^*−/−*^ male mice were infected with VSV by intraperitoneal injection. ELISA was used to measure the cytokine production of serum. The VSV titers in the brain, lung, spleen, and liver were determined by plaque assays in Vero cells. For the survival experiments, mice were monitored for survival after intraperitoneal injection of VSV (1 × 10^8^ pfu/mouse).

For intracerebral infection, 8-week-old *Usp18*^*+/+*^ and *Usp18*^*−/−*^ male mice were anesthetized and placed on a stereotaxic apparatus for lateral ventricle injection of VSV (1000 PFU) at a rate of 0.2 µl/min. The coordinates were as follows: 0.4 mm posterior to the bregma, 1 mm lateral, 2.2 mm deep.

### Lung histology

Lungs from control or virus-infected mice were dissected, fixed in 10% phosphate-buffered formalin, embedded into paraffin, sectioned, stained with hematoxylin and eosin solution, and examined by light microscopy for histological changes.

### Generation of knockout cells by CRISPR/Cas9 technology

HeLa cells with the knockout of MAVS were generated by a plenti-CRISPR/Cas9-v2 system, and the sequences of target MAVS-sgRNAs are as follows: sense: GATTGCGGCAGATATACTTAT, antisense: ATAAGTATATCTGCCGCAATC. The isolated single clonal knockout cells survived after puromycin killing were confirmed with western blotting. HeLa cells with the knockout of MAVS are readily available upon request to the corresponding authors.

### Statistical analysis and reproducibility

All experiments were performed for at least two times. All data were processed with Microsoft Excel and GraphPad Prism 8.0 and are presented as mean ± SD of one representative experiment. Statistical significance between groups was determined by two-tailed Student’s *t* test, with a *p* value < 0.05 considered statistically significant. For mouse survival studies, Kaplan–Meier survival curves were generated and analyzed for statistical significance with GraphPad Prism 8.0.

### Reporting summary

Further information on research design is available in the Nature Research Reporting Summary linked to this article.

## Supplementary information

Supplementary Information

Reporting Summary

## Data Availability

All data are provided in the article and its Supplementary files or from the corresponding author upon reasonable request. Source data are provided with this paper.

## Source data

Source data
