# Supplementary material for: USP18 positively regulates innate antiviral immunity by promoting K63-linked polyubiquitination of MAVS
Source: Nat Commun. 2021 May 20;12:2970. doi: 10.1038/s41467-021-23219-4 (PMC8137702; doi:10.1038/s41467-021-23219-4)
Supplement: Supplementary file 1 — Supplementary Information [file 41467_2021_23219_MOESM1_ESM.pdf]

Supplementary Information for

USP18 positively regulates innate antiviral immunity through promoting the K63-linked polyubiquitination of MAVS

Jinxiu Hou[1], Lulu Han[1], Ze Zhao[2], Huiqing Liu[2], Lei Zhang[1], Chunhong Ma[1], Fan Yi[2], Bingyu Liu[1], Yi Zheng[1, *], Chengjiang Gao[1,3,*]

[1]Key Laboratory of Infection and Immunity of Shandong Province & Department of Immunology, School of Basic Medical Sciences, Shandong University, Jinan, Shandong 250012, P. R. China; [2]Department of Pharmacology, School of Basic Medical Sciences, Shandong University, Jinan, Shandong 250012, P. R. China; [3]Lead Contact

[*]Corresponding author: Dr. Chengjiang Gao

E-mail: cgao@sdu.edu.cn

Or Dr. Yi Zheng

E-mail: zhengyiabc2011@sdu.edu.cn

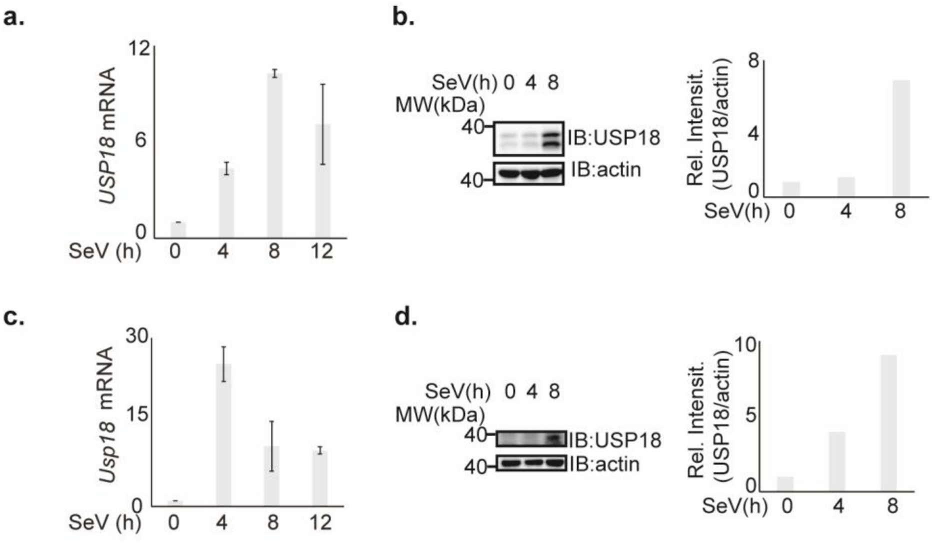

Supplementary Figure 1. **SeV infection induces the mRNA and protein level of USP18.** a,b). qRT-PCR analysis (a) of *USP18* expression and immunoblot analysis (b) of USP18 protein level in THP-1 cells infected with SeV for indicated time points. c,d). qRT-PCR analysis (c) of *Usp18* expression and immunoblot analysis (d) of USP18 protein level in peritoneal macrophages infected with SeV for indicated times. Band intensity of USP18 and actin blots were quantified in ImageJ, and the intensities of USP18 were normalized to actin. Representative data were collected and expressed as mean ± SD from three independent experiments (a and c).

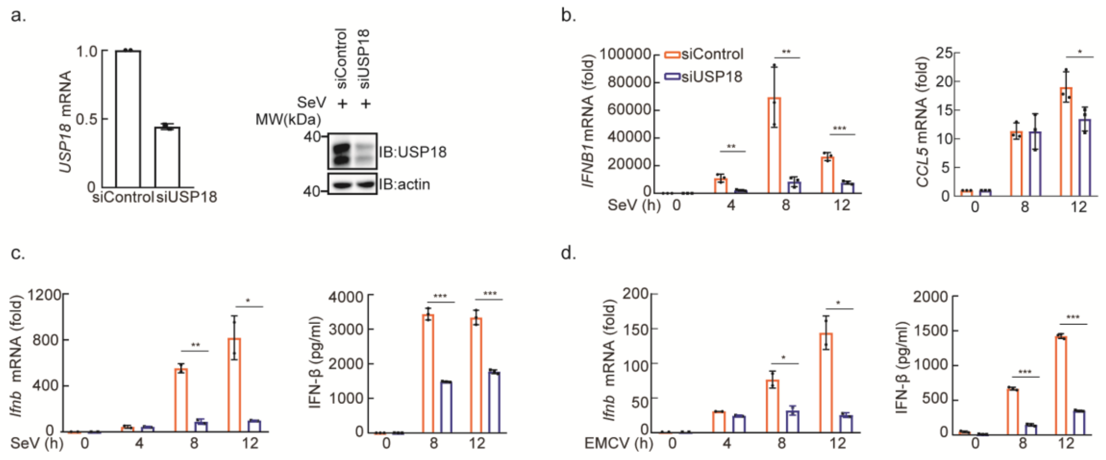

Supplementary Figure 2. USP18 regulates IFN-β signaling. a) qPCR (left) and western blot (right) analysis of siRNA-mediated knockdown of USP18 in THP-1 cells. b) qRT-PCR analysis of *IFNB* (left) and *CCL5* (right) analysis from THP-1 cells transfected with the control siRNA or siRNA against *USP18* followed by infection with SeV for indicated time points. (mean ± SD, two-tailed t-test, **p=0.0065, **p=0.0087, ***p=0.0005, *p=0.0456) c,d) qRT-PCR analysis of *Ifnb* (left) and ELISA analysis of IFN-β (right) from mouse primary peritoneal macrophages transfected with the control siRNA or siRNA against *Usp18* followed by infection with SeV(c) or EMCV (d) for indicated time points. For c, mean ± SD, two-tailed t-test, **p=0.0050, *p=0.0333, ***p<0.001, ***p<0.001. For d, mean ± SD, two-tailed t-test, *p=0.0153 *p=0.0453, ***p<0.001, ***p<0.001. Representative data were collected and expressed as mean ± SD from three independent experiments.

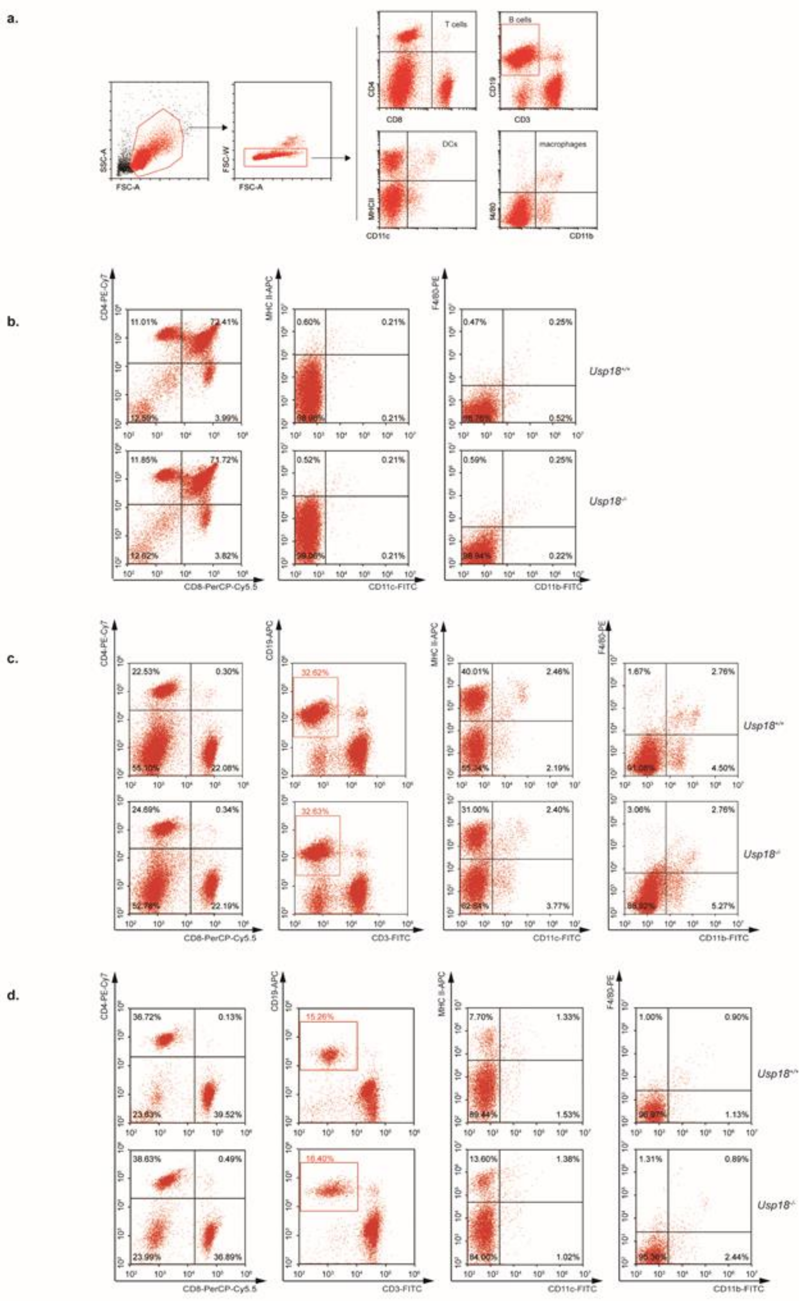

Supplementary Figure 3. Analysis of *Usp18*$^{+/+}$ and *Usp18*$^{–/–}$ mice. a) Gating strategy to determine the percentage of immune cells that differentiate into T cells, B cells, DCs, and macrophages, respectively. b, c, and d) Flow cytometry analysis of immune cells and quantitative data in the thymus (a), spleen (b), and peripheral lymph nodes (c) from *Usp18*$^{+/+}$ and *Usp18*$^{-/-}$ mice (n=3).

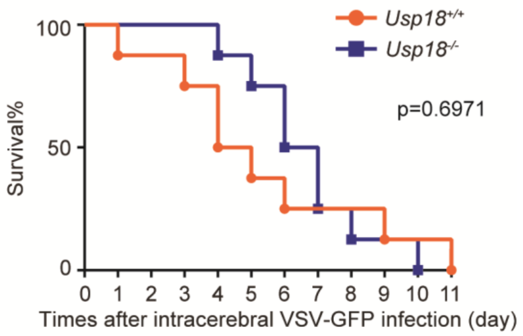

Supplementary Figure 4. Survival (Kaplan–Meier curves) with log rank test [Mantel-Cox] of *Usp18*$^{+/+}$ and *Usp18*$^{-/-}$ mice (eight per group) injected intracerebrally with VSV (1×10^3 PFU per mouse).

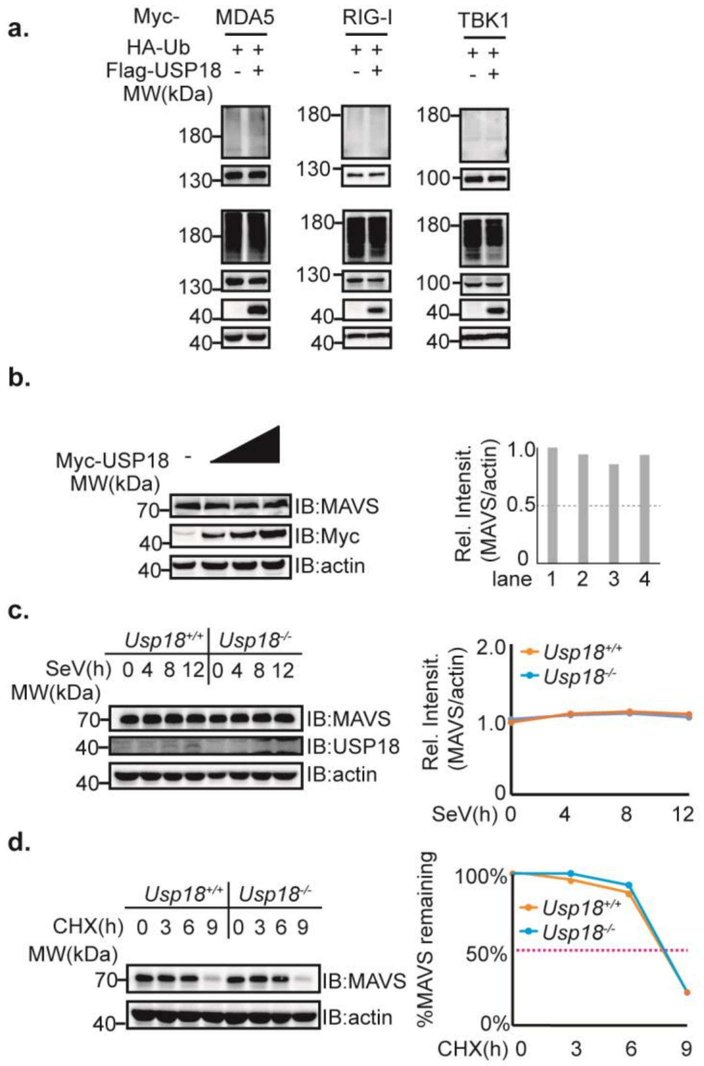

Supplementary Figure 5. USP18 does not regulate the ubiquitination of RIG-I, MDA5, and TBK1 as well as the stability of MAVS. a) Immunoprecipitation analysis of ubiquitination of signaling molecules in HEK293T cells transfected with the plasmids expressing Myc-RIG-I, MDA5, or TBK1, HA-Ub, and Flag vector or Flag-USP18. b) Left panel: Immunoblot analysis of protein level of endogenous MAVS in HEK293T cells transfected with Myc-USP18 in different dosages (0 μg, 1μg, 2μg, and 3μg). Right panel: Band intensity of MAVS and actin blots were quantified in ImageJ, and the intensities of MAVS were normalized to actin. c) Left panel: Immunoblot analysis of protein level of endogenous MAVS in *Usp18*$^{+/+}$ and *Usp18*$^{-/-}$ MEFs infected with SeV for 0-12h and harvested at indicated times. Right panel: Band intensity of MAVS and actin blots were quantified in ImageJ, and the intensities of MAVS were normalized to actin. d) Left panel: Immunoblot analysis of

protein level of endogenous MAVS in *Usp18*$^{+/+}$ and *Usp18*$^{-/-}$ MEFs treated with 100 ug/ml Cycloheximide (CHX) for 0-9h and harvested at indicated times. Right panel: Band intensity of MAVS and actin blots were quantified in ImageJ, and the intensities of MAVS were normalized to actin.

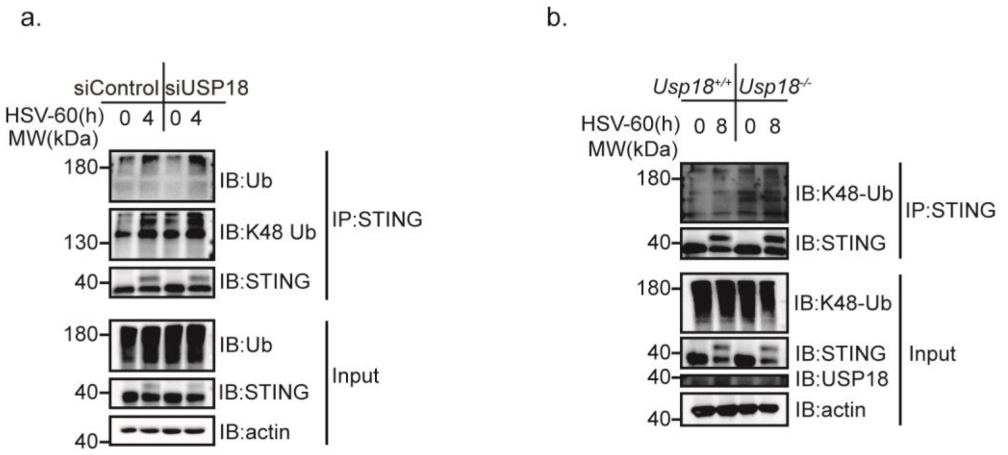

Supplementary Figure 6. USP18 regulates the K48-linked polyubiquitination of STING. a). Immunoblot analysis of ubiquitination of endogenous STING in RAW264.7 cells transfected with siControl or siUSP18 followed by stimulation with HSV-60 for indicated time points. b). Immunoblot analysis of ubiquitination of endogenous STING in *Usp18*$^{+/+}$ and *Usp18*$^{-/-}$ MEFs stimulated with HSV-60 for indicated time points.

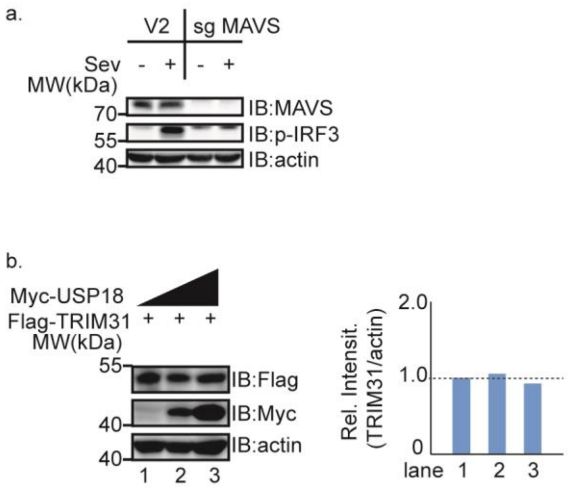

Supplementary Figure 7. Characterization of MAVS KO HeLa cells, and USP18 does not regulate the stability of TRIM31. a) Immunoblot analysis of MAVS, p-IRF3, and actin in lysates of HeLa cells with lentiCRISPRv2 or sg MAVS subjected to infection with SeV. b) Left panel: immunoblot analysis of protein level of Flag-TRIM31 in HEK293T cells transfected with Flag-TRIM31 (1μg) in fixed amount and Myc-USP18 in different dosages (0μg, 1μg, and 2μg). Right panel: Band intensity of Flag-TRIM31 and actin blots were quantified in ImageJ, and the intensities of Flag-TRIM31 were normalized to actin.

Supplementary Table 1. Sequences of PCR primers used in this study.

**Oligonucleotides**	**Sequences**
mouse *Ifnb1*-F	5'-ATGAGTGGTGGTTGCAGGC-3'
mouse *Ifnb1*-R	5'-TGACCTTTCAAATGCAGTAGA-3'
mouse *Ccl5*-F	5'-TCACCATATGGCTCGGACACCAC-3'
mouse *Ccl5*-R	5'-TTGGCACACACTTGGCGGTTC-3'
mouse actin-F	5'-CCACACCCGCCACCAGTTCG-3'
mouse actin-R	5'-TACAGCCCGGGGAGCATCGT-3'
mouse *Usp18*-F	5'- GAGCTTGGATTTCAGTCAGGTT-3'
mouse *Usp18*-R	5'- CATTGAAGCAGAACCACTTTCC-3'
human IFNB1-F	5'-CAACAAGTGTCTCCTCCAAAT-3'
human IFNB1-R	5'-TCTCCTCAGGGATGTCAAAG-3'
human ACTIN-F	5'-GGAAATCGTGCGTGACATTAA-3'
human ACTIN-R	5'-AGGAAGGAAGGCTGGAAGAG-3'
human *CCL5*-F	5'- CCTGCTGCTTTGCCTACATTGC -3'
human *CCL5*-R	5'- ACACACTTGGCGGTTCTTTCGG -3'
human *USP18*-F	5'- CCTGCTGCTTTGCCTACATTGC -3'
human *USP18*-R	5'- CGAACACCTGAATCAAGGAGTTA -3'
