# Supplementary material for: USP18 positively regulates innate antiviral immunity by promoting K63-linked polyubiquitination of MAVS
Source: Nat Commun. 2021 May 20;12:2970. doi: 10.1038/s41467-021-23219-4 (PMC8137702; doi:10.1038/s41467-021-23219-4)
Supplement: Supplementary file 2 — Reporting Summary [file 41467_2021_23219_MOESM2_ESM.pdf]

nature research

Corresponding author(s): Chengjiang Gao

Last updated by author(s): Mar 4, 2021

# Reporting Summary

Nature Research wishes to improve the reproducibility of the work that we publish. This form provides structure for consistency and transparency in reporting. For further information on Nature Research policies, see our Editorial Policies and the Editorial Policy Checklist.

## Statistics

For all statistical analyses, confirm that the following items are present in the figure legend, table legend, main text, or Methods section.

| n/a | Confirmed | |
|---|---|---|
| ☐ | ☒ | The exact sample size (*n*) for each experimental group/condition, given as a discrete number and unit of measurement |
| ☐ | ☒ | A statement on whether measurements were taken from distinct samples or whether the same sample was measured repeatedly |
| ☐ | ☒ | The statistical test(s) used AND whether they are one- or two-sided<br>*Only common tests should be described solely by name; describe more complex techniques in the Methods section.* |
| ☒ | ☐ | A description of all covariates tested |
| ☒ | ☐ | A description of any assumptions or corrections, such as tests of normality and adjustment for multiple comparisons |
| ☐ | ☒ | A full description of the statistical parameters including central tendency (e.g. means) or other basic estimates (e.g. regression coefficient) AND variation (e.g. standard deviation) or associated estimates of uncertainty (e.g. confidence intervals) |
| ☐ | ☒ | For null hypothesis testing, the test statistic (e.g. *F*, *t*, *r*) with confidence intervals, effect sizes, degrees of freedom and *P* value noted<br>*Give P values as exact values whenever suitable.* |
| ☒ | ☐ | For Bayesian analysis, information on the choice of priors and Markov chain Monte Carlo settings |
| ☒ | ☐ | For hierarchical and complex designs, identification of the appropriate level for tests and full reporting of outcomes |
| ☒ | ☐ | Estimates of effect sizes (e.g. Cohen's *d*, Pearson's *r*), indicating how they were calculated |

*Our web collection on statistics for biologists contains articles on many of the points above.*

## Software and code

Policy information about availability of computer code

| | |
|---|---|
| Data collection | Microsoft Excel 2016, Zeiss Zen, qPCRsoft 4.0, SageCapture TM, CytExpert |
| Data analysis | Microsoft Excel 2016, Zeiss Zen, ImageJ, GraphPad Prism 8, Adobe Photoshop, Adobe Illustrator |

For manuscripts utilizing custom algorithms or software that are central to the research but not yet described in published literature, software must be made available to editors and reviewers. We strongly encourage code deposition in a community repository (e.g. GitHub). See the Nature Research guidelines for submitting code & software for further information.

## Data

Policy information about availability of data

All manuscripts must include a data availability statement. This statement should provide the following information, where applicable:

- Accession codes, unique identifiers, or web links for publicly available datasets
- A list of figures that have associated raw data
- A description of any restrictions on data availability

The original data that support the findings of this study are available from the corresponding author upon reasonable request. Supplementary figures are available in the Supplementary Information. Source data are provided with this paper.

# Field-specific reporting

# Life sciences study design

All studies must disclose on these points even when the disclosure is negative.

| | |
|---|---|
| Sample size | Pilot studies were used for estimation of the sample size required to ensure adequate power. |
| Data exclusions | No exclusion of data points or mice was used. |
| Replication | Each experiment has been repeated for at least two times with similar results. The figures included in the paper are the representative results. |
| Randomization | No statistical methods were used for randomization. For in vitro experiments, mouse peritoneal macrophages were isolated from randomly chosen wild-type or KO mice. For in vivo experiments, wild-type or KO mice were randomly allocated into experimental groups. |
| Blinding | The investigators were blinded during collection and data analysis if possible, such as RT-PCR, ELISA and confocal microscopy. |

# Reporting for specific materials, systems and methods

We require information from authors about some types of materials, experimental systems and methods used in many studies. Here, indicate whether each material, system or method listed is relevant to your study. If you are not sure if a list item applies to your research, read the appropriate section before selecting a response.

## Materials & experimental systems

| n/a | Involved in the study | |
|---|---|---|
| ☐ | ☒ | Antibodies |
| ☐ | ☒ | Eukaryotic cell lines |
| ☒ | ☐ | Palaeontology and archaeology |
| ☐ | ☒ | Animals and other organisms |
| ☒ | ☐ | Human research participants |
| ☒ | ☐ | Clinical data |
| ☒ | ☐ | Dual use research of concern |

## Methods

| n/a | Involved in the study | |
|---|---|---|
| ☒ | ☐ | ChIP-seq |
| ☐ | ☒ | Flow cytometry |
| ☒ | ☐ | MRI-based neuroimaging |

## Antibodies

| | |
|---|---|
| Antibodies used | Rabbit anti-HA Ab (Cat#600-401-384)was from Rockland; Rabbit anti-USP18 (D4E7, Cat#4813), rabbit anti-GFP(D5.1)XP(Cat#2956), rabbit anti-DYKDDDDK Tag (D6W5B,Cat#14793), mouse anti-ubiquitin (P4D1,Cat#3936), rabbit anti-K48 linkage specific ubiquitin (D9D5,Cat#5621), rabbit anti-K63 linkage specific ubiquitin (D7A11,Cat#8081), rabbit anti-IRF3 (D83B9,Cat#4302), rabbit anti-pIRF3 (4D46,Cat#4947), rabbit anti-TBK1 (3031S,Cat#3504), rabbit anti-pTBK1 (D52C2,Cat#5483), and normal rabbit IgG Abs (Cat#2729) were from Cell Signaling Technology; Mouse anti-MAVS(Cat#SC-365333,SC-166853), mouse anti-α tubulin(Cat#SC-5286), and normal mouse IgG Abs(Cat#SC-2025), VDAC1(B-6) sc-390996 were from Santa Cruz Biotechnology; Mouse anti-actin(Cat#66009), rabbit-anti-STING(Cat#19851-1-AP), and rabbit-anti-human TRIM31 Abs (12543-1-AP) were from proteintech; Mouse anti-Flag M2(Cat#A2220) and rabbit anti-mouse TRIM31 Abs (Cat#AV34717) were from Sigma Aldrich; Mouse anti-Myc (9E10,Cat#TA150121) Ab was from Origene; Rabbit anti-TOM20 Ab (Cat#A6774) and rabbit anti-USP18 Ab (Cat#A16739) was from Abclonal; Rabbit anti-Sendai virus Ab (Cat#PD029) was from MBL. |
| Validation | Rabbit anti-USP18 (D4E7, Cat#4813) validated in human for WB, Ribosome biogenesis restricts innate immune responses to virus infection and DNA. Elife 2019<br>rabbit anti-GFP(D5.1)XP(Cat#2956) validated for WB, A salt-induced kinase is required for the metabolic regulation of sleep. PLoS Biol 2020<br>rabbit anti-DYKDDDDK Tag (D6W5B,Cat#14793) validated for IFA , Sorting Nexin 27 Regulates the Lysosomal Degradation of Aquaporin-2 Protein in the Kidney Collecting Duct. Cells 2020<br>mouse anti-ubiquitin (P4D1,Cat#3936) validated for WB, LncRNA DILA1 inhibits Cyclin D1 degradation and contributes to tamoxifen resistance in breast cancer. Nat Commun 2020<br>rabbit anti-K48 linkage specific ubiquitin (D9D5,Cat#5621) validated for WB, TRAF3IP3 negatively regulates cytosolic RNA induced anti-viral signaling by promoting TBK1 K48 ubiquitination. Nat Commun 2020<br>rabbit anti-K63 linkage specific ubiquitin (D7A11,Cat#8081) validated for WB, Cardiomyocyte Contractility and Autophagy in a Premature Senescence Model of Cardiac Aging. Oxid Med Cell Longev 2020<br>rabbit anti-IRF3 (D83B9,Cat#4302) validated for WB, SKIL facilitates tumorigenesis and immune escape of NSCLC via upregulating TAZ/autophagy axis. Cell Death Dis 2020<br>rabbit anti-pIRF3 (4D4G,Cat#4947) validated for WB, G3BP1 controls the senescence-associated secretome and its impact on cancer progression. Nat Commun 2020<br>rabbit anti-TBK1 (D1B4,Cat#3504) validated for WB, SKIL facilitates tumorigenesis and immune escape of NSCLC via upregulating TAZ/autophagy axis. Cell Death Dis 2020<br>rabbit anti-pTBK1 (D52C2,Cat#5483) validated for WB, SKIL facilitates tumorigenesis and immune escape of NSCLC via upregulating TAZ/autophagy axis. Cell Death Dis 2020<br>Mouse anti-MAVS (SC-166853) validated for WB, Kok, K.H., et al. 2011. The double-stranded RNA-binding protein PACT functions as a |

cellular activator of RIG-I to facilitate innate antiviral response. Cell Host Microbe 9: 299-309.
Mouse anti-MAVS (Cat#SC-36533) validated for WB, Liuyu, T., et al. 2019. Induction of OTUD4 by viral infection promotes antiviral responses through deubiquitinating and stabilizing MAVS. Cell Res. 29: 67-79.
Mouse anti-α tubulin(Cat#SC-5286)validated for WB, Golub, T., et al. 2002. The Ewing's sarcoma oncoprotein EWS/Fli induces a p53-dependent growth arrest in primary human fibroblasts. Cancer Cell 1: 393-401.
Mouse anti-VDAC1 validated for WB , Oroxylin A induces dissociation of hexokinase II from the mitochondria and inhibits glycolysis by SIRT3-mediated deacetylation of cyclophilin D in breast carcinoma. Cell Death Dis 2013
Mouse anti-actin (Cat#66009) validated for WB, Cell-autonomous adiposity through increased cell surface GLUT4 due to ankyrin-B deficiency. Proc Natl Acad Sci U S A
Rabbit-anti-STING(Cat#19851-1-AP) validated for WB and IFA, Autophagy induction via STING trafficking is a primordial function of the cGAS pathway. Nature
Rabbit-anti-human TRIM31 Abs (12543-1-AP) validated for WB, Tripartite motif 31 promotes resistance to anoikis of hepatocarcinoma cells through regulation of p53-AMPK axis. Exp Cell Res
Rabbit anti-mouse TRIM31 Abs (Cat#AV34717) validated for WB, Control of antiviral innate immune response by protein geranylgeranylation. Science advances
Rabbit anti-TOM20 Ab (Cat#A6774) validated for WB and IFA, Fructose-1,6-Bisphosphatase 2 Inhibits Sarcoma Progression by Restraining Mitochondrial Biogenesis. Cell Metabolism
Rabbit anti-USP18 Ab (Cat#A16739) tested by ABclonal, we validated through comparing USP18 WT and KO mice lysate.
SEC61B antibody from Abclonal have been KO validated for WB by the company.
Rabbit anti-Sendai virus Ab (Cat#PD029) validated for WB, Hasan M et al. Trex1 regulates lysosomal biogenesis and interferon-independent activation of antiviral genes. Nat Immunol. 14, 61-71 (2013) (PMID:23160154)

# Eukaryotic cell lines

Policy information about cell lines

| | |
|---|---|
| Cell line source(s) | HEK293T, HeLa, THP-1, and RAW264.7 cells were obtained from American Type Culture Collection. |
| Authentication | None of the cell lines were authenticated. |
| Mycoplasma contamination | We tested and confirmed that all used cell lines are mycoplasma negative. |
| Commonly misidentified lines (See ICLAC register) | No commonly misidentified lines were used. |

# Animals and other organisms

Policy information about studies involving animals; ARRIVE guidelines recommended for reporting animal research

| | |
|---|---|
| Laboratory animals | Usp18+/− mice were a mixed Sv129-C57BL/6 background. Usp18+/− mice were crossed to obtain Usp18+/+ and Usp18−/− mice. All mice were housed in the specific pathogen-free animal facility with 40–70% humidity and daily cycles of 12 h of light at 23 °C and 12 h of dark at 21 °C at Shandong University and all animal experiments were under protocols approved by the Institutional Animal Care and Use Committee of Shandong University. Eight to twelve weeks old littermate mice were used for the experiments. |
| Wild animals | No wild animals were used. |
| Field-collected samples | No field-collected samples were used. |
| Ethics oversight | All animal experiments were under protocols approved by the Institutional Animal Care and Use Committee of Shandong University. |

Note that full information on the approval of the study protocol must also be provided in the manuscript.

# Flow Cytometry

## Plots

Confirm that:

- [x] The axis labels state the marker and fluorochrome used (e.g. CD4-FITC).
- [x] The axis scales are clearly visible. Include numbers along axes only for bottom left plot of group (a 'group' is an analysis of identical markers).
- [x] All plots are contour plots with outliers or pseudocolor plots.
- [x] A numerical value for number of cells or percentage (with statistics) is provided.

## Methodology

| | |
|---|---|
| Sample preparation | The 6-8 weeks old USP18+/+ and USP18-/- mice were sacrificed, and lymphoid cells from the thymus, spleen, and lymph node were incubated with fluorochrome conjugated antibodies. |
| Instrument | BECKMAN COULTER CytoFLEX |

| | |
|---|---|
| Software | CytExpert |
| Cell population abundance | Expressed as the frequency of the selected population. |
| Gating strategy | Forward versus side scatter (FSC vs SSC) gating is first used to remove debris. Next, two parameter density plots by using the indicated marker are used as the subsetting gating, in which the FMO controls were also set up. |

☒ Tick this box to confirm that a figure exemplifying the gating strategy is provided in the Supplementary Information.
